# Enabling the Development and Deployment of Next Generation Point-of-Care Diagnostics

**DOI:** 10.1371/journal.pntd.0003676

**Published:** 2015-05-14

**Authors:** Ratmir Derda, Jesse Gitaka, Catherine M. Klapperich, Charles R. Mace, Ashok A. Kumar, Marya Lieberman, Jacqueline C. Linnes, Joerg Jores, Johnson Nasimolo, Joseph Ndung’u, Evans Taracha, Abigail Weaver, Douglas B. Weibel, Thomas M. Kariuki, Paul Yager

**Affiliations:** 1 Department of Chemistry and Alberta Glycomics Centre, University of Alberta, Edmonton, Alberta, Canada; 2 Department of Clinical Medicine, Mount Kenya University, Thika, Kenya; 3 Department of Biomedical Engineering and Center for Future Technologies in Cancer Care, Boston University, Boston, Massachusetts, United States of America; 4 Diagnostics For All, Cambridge, Massachusetts, Unites States of America; 5 Department of Chemistry, Tufts University, Medford, Massachusetts, United States of America; 6 School of Engineering and Applied Sciences, Harvard University, Cambridge, Massachusetts, United States of America; 7 Department of Chemistry and Biochemistry, University of Notre Dame, Notre Dame, Indiana, United States of America; 8 International Livestock Research Institute, Nairobi, Kenya; 9 Department of Veterinary Anatomy and Physiology, University of Nairobi, Nairobi, Kenya; 10 Foundation for Innovative New Diagnostics, Geneva, Switzerland; 11 Institute of Primate Research, National Museums of Kenya, Nairobi, Kenya; 12 Departments of Biochemistry, Biomedical Engineering, and Chemistry, University of Wisconsin-Madison, Madison, Wisconsin, United States of America; 13 Department of Bioengineering, University of Washington, Seattle, Washington, United States of America; Centers for Disease Control and Prevention, UNITED STATES

## Introduction

### About the Conference

The workshop highlighted emerging diagnostic technologies that had been presented at the site and assembled and/or tested by the attendees during the hands-on sessions. The venue attracted more than 30 presenters and 126 participants (39 international and 87 African). Specifically, African participants were from Kenya (73), Uganda (7), Tanzania (3), Ethiopia (1), Rwanda (1), Zimbabwe (1), and South Africa (1). African participants were affiliated with universities (41 from Nairobi, Kenyatta, Makerere, Egerton, Aga Khan, Strathmore, and others), hospitals and ministries, (5) or research institutes (38) such as the Institute of Primate Research (IPR), The Kenya Medical Research Institute (KEMRI), the International Livestock Research Institute (ILRI), The International Centre of Insect Physiology and Ecology (ICIPE), and the Tsetse & Trypanosomiasis Research Institute (TTRI/EGFAR). More detailed summary of the participants can be found on page 75 of the conference program (provided as a supplementary file). The workshop program consisted of three days of presentations, including a poster session and an additional two full days of hands-on demonstrations of devices that have a potential to become next generation diagnostic platforms. Reports on the development of many of these devices have been published, and their descriptions can be found in references [1–17] and Table 1. Importantly, however, the majority of the technologies presented had not been tested outside of a research laboratory setting. Out of 18 point-of-care (POC) devices brought to the site, 12 were assembled on-site, and 4 were manufactured from raw components (Table 1) in a self-made production facility, which was set up in a laboratory at the IPR (http://www.primateresearch.org/). A full description of the devices, list of conference presenters, attendees, and abstracts can be found in the conference program (provided as a supplementary file S1 Text); additional information, such as the videos of demonstrations and talks can be found at: http://www.glycomicscentre.ca/conferences/past-workshops/ and https://www.youtube.com/watch?v=cYTs3FoOOo4.

**Table 1:** Devices demonstrated at the workshop.

Device	Lab/Group (University)	Target/Capability	Assembled On-Site	References
Webcam microscope	Hackteria	Digital microscopy	Y	[18]
DIY laser cutter	Hackteria	Fabrication	Y	[19]
Paper-based device	Diagnostics for all	Liver function		[16,20,21]
Aqueous multiphase systems & egg beater centrifuge	Whitesides (Harvard)	Hematology	Y	[1,13,22–24]
Paper millifluidic test card	Lieberman (Notre Dame)	Detection of fake pharmaceuticals	Y	[17]
2DPN for controlled flow	Yager (U. Washington)	ELISA	Y	[5,7]
Microfluidic hematology analyzer	Morgan (U. Southampton)	Complete blood count	Y	[8,25]
3D paper devices	Martinez (Cal Poly)	Urinanalysis	Y*	[12]
Polymer-based color tunable materials	Serpe (U. Alberta)	Glucose	Y	[26]
Cell sorting with pegs	Tegenfeldt (Lund University)	Parasite detection	Y	[9]
BacChip	Weibel (U. Wisconsin)	Bacterial infections		[10,27]
DNA amplification by destabilization	Gibbs-Davis (U. Alberta)	Nucleic acid detection	Y	[14,28]
Portable bacteria cultures	Derda (U. Alberta)	Environmental monitoring	Y*	[11]
Adherio	Klapperich/Gomez-Marquez (Boston University/MIT)	TB Drug Adherence monitoring	Y	
Shrink-wrap microfluidics	Khine (UC Irvine)	Fabrication of microfluidics	Y*	[4]
Electrophoretic focusing chip	Cooper (U. Glasgow)	Trypanosomiasis		[29]
Plasma separation on chip	Shu (Heriot-Watt)	Sample preparation		
Multiwell plates	Carrilho (U. Sao Paulo)	Multiwell analysis	Y*	[2]

(*Devices were produced on site)

### Genesis of This Document

During the conference, it became clear that ideas generated via interactions between North American, South American, European, and African participants could be instructive for the wider community of scientists working on POC diagnostics and the diseases that disproportionately affect low- and middle-income countries. At the crux of this document is our belief that change is only possible by building partnerships with the health science and health care systems in these countries. The demand for low cost, POC diagnostics exists, but the commercialization mechanisms needed to see them through to distribution and sustainability are, for the most part, inefficient or nonexistent. Only by building and reinforcing these systems will POC diagnostics find their way into the hands of the health care providers or clinicians who need solutions. This document shares ideas that we developed during in-person discussions and continuing correspondence between the authors from the 2012 workshop until 2015. We focus on applications in East Africa; however, we anticipate that many of these recommendations can be applied generally. We suggest solutions to the most commonly identified “broken” links along the value chain that connects ideas in the research lab with doctors, health care workers, and patients in the clinic. We anticipate that this document will serve as a renewed call to action for the development and successful deployment of POC diagnostics in low resource settings, and we address these comments to funders, governments, researchers, clinicians, and, most importantly, policy makers.

### The Continuing Need for POC Diagnostics in Low Resource Settings

As the developing world strives to meet the Millennium Development Goals (MDGs) and improve quality of life [30], it will be critical to address the current heavy burden of diseases. In the therapeutic process, correct and timely diagnosis is the pillar on which treatment, follow-up, and public control measures hinge. Examples of success exist. The introduction of rapid tests for HIV has contributed greatly to widespread testing, demand for downstream services, prompt diagnosis, treatment, follow-up, and behavior change [31,32].

An example of partial success is the development and commercialization of rapid diagnostic tests (RDTs) for malaria. Malaria RDTs have been a critical tool for health care workers operating in remote locations lacking access to electricity, experienced microscopists, or other resources required to diagnose and treat patients with confidence [33]. These tests have been used to manage febrile (i.e., symptomatic) children in countries with endemic malaria [34]. Despite this success, outstanding challenges still exist in malaria diagnostics. Although the sensitivity of existing RDTs are generally able to detect malaria in a febrile patient—an important step in effective programs to manage the disease—the sensitivity is not always sufficient to detect lower levels of infections and asymptomatic carriers, which are both important in eradication campaigns [35,36]. Many malaria RDTs do not detect mixed infections and, depending on the antigenic marker used, may not be effective in monitoring responses to therapy [37,38]. A further obstacle to adoption is the attitude of health care workers towards the use of these tests [39]. Recent work in Senegal provides a model of training and implementation that may be able to overcome some of these challenges [40]. Additionally, the World Health Organization (WHO), in collaboration with the Foundation for Innovative New Diagnostics (FIND) and others, has undertaken a rigorous evaluation of the multitude of RDTs on the market; this assessment will provide users with the information required to choose the best tests available for their needs [41].

In addition to improving existing RDTs, new tests must be developed to provide options to assist in the monitoring and control of emerging diseases or those considered difficult to diagnose at the POC. Tuberculosis is an excellent example of this category of disease. One third of the world population is infected with tuberculosis (TB), approximately 9 million persons are infected each year, and there are approximately 1.2 million deaths annually [42]. Diagnostics for TB, such as Cepheid’s GeneXpert, are an excellent display of advances in diagnostic technologies but are still too expensive and bulky for POC use [43]. The 2014–2015 Ebola outbreak in West Africa and the subsequent challenges in diagnosing and controlling the disease is a classical gap that could be bridged by POC diagnostics. Timely and reliable diagnosis of cases in primary health care facilities might have altered the course of the outbreak, saving many lives with minimal disruption of the day-to-day life in the affected areas (Dhillon, 2014 #172). These POC diagnostics would not only diagnose Ebola but in a multiplex design be able to rule out other febrile infections prevalent in the region. Other priority areas include neglected tropical diseases that afflict over 1,000,000,000 of the world’s poor [44], and non-HIV sexually transmitted infections (e.g., gonorrhea, chlamydia, and syphilis). Additionally, the rising burden of noncommunicable diseases in low- and middle-income countries (e.g., hypertension, diabetes, and cancer [45]) amplifies the need for simple tests that can diagnose the disease and its complications, track treatment progress milestones, and monitor serum drug levels. As the demographics of disease change, future tests will play a greater role in preventive medicine rather than curative medicine. As efforts accelerate towards eliminating diseases, including malaria, simple tests will be critical in monitoring epidemiological trends in communities (e.g., gametocytemia prevalence) [35]. All disease control efforts require effective and reliable diagnostics in order to carry out interventions that can ultimately succeed.

### Definitions

To properly frame the problems and proposed solutions (Table 2), we define key concepts below.

**Table 2:** Barriers and potential solutions to advancing POC diagnostics research.

Current Barriers	Potential Solutions
•Disconnect between developers and end users	•Begin collaborations early (i.e., needs and design process)
	•Publish in open-access journals to make information available to both users and developers
•Lack of funding for international collaboration	•Advocate for multidisciplinary research funding
	•Engage in policy discussions and set priorities
	•Establish funds for exchange programs
•Funding priorities do not always reflect local priorities in developing countries	•Direct partnerships and assessments
	•Create new metrics to demonstrate impact of POC diagnostics on health costs & quality
•Costs of intellectual property (IP)	•Establish protocols for transferring technology between countries
•Resistance to interdisciplinary work in some countries	•Collaboration and modeling interdisciplinary work
•Distrust of Western researchers	•High quality control standards
	•Equal partnerships (local co-PI)
•Inertia to continue existing practices	•Develop and update health curricula to reflect advances in POC diagnostics
	•Refresher courses to update users on new devices
	•Engage with mass media to disseminate advances
	•Dedicate online resources to aid in the use and interpretation of POC diagnostics
•Failure of prototypes to become products	•Design with sustainability in mind
	•Understand supply chains
	•Use proper controls
	•User-centered design

#### Point-of-care diagnostics

We define POC diagnostics as small, portable devices capable of detecting the presence or absence of a disease-causing agent, a disease, or quantifying the severity or a change in severity of a disease. A broader subset of POC tests also includes portable tests for quality control; for example, prototypes of the devices for detection of counterfeit pharmaceuticals were displayed and tested at this conference [17]. WHO and others [46] have outlined the functional and performance specifications for “ideal” POC diagnostic assays [47]; we will not reiterate those lists here. We do include in our definition stand-alone, one-time use devices that require no additional instrumentation, minimally-instrumented devices, and devices that include portable and easy-to-use instrumentation (e.g., cell phone-based approaches to telemedicine [48,49]).

#### Value chain

**Fig 1:**
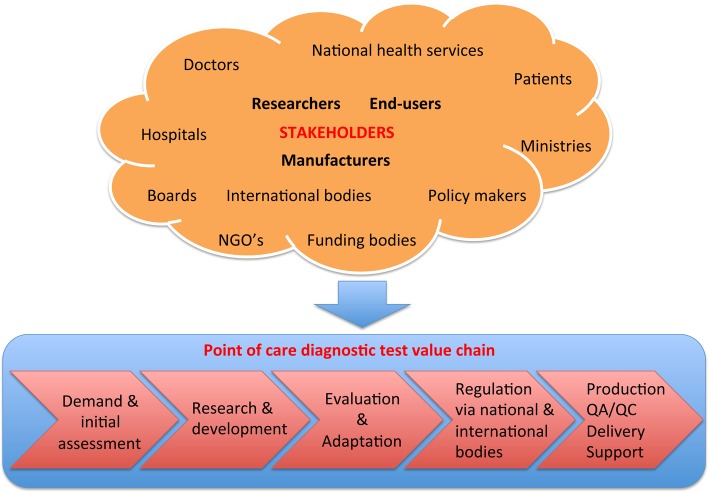
Value chain of point-of-care diagnostics. The top part displays the stakeholders that are involved in the development, commercialization, roll out, and integration of point-of-care diagnostic tests into control and surveillance programs

Over the course of the workshop, it became clear that scientists involved in developing or using assays felt confident in their ability to define and tackle technical problems in device and assay design. However, we felt less confident as a group in our ability to identify problems along the entire chain of events between the design and development of a new device and its adoption by an end user. It is useful to define this entire process as the POC diagnostic “value chain” (Fig 1). Each link in this chain represents a potential point of failure for the entire progression from research and development to the deployment of a product that becomes part of patient care [50,51]. This chain includes: (i) assessments of clinical, market, and end user needs; (ii) determination of demand for the product (in part) through marketing, procurement, and partnership with local governmental and nongovernmental organizations; (iii) design and development of diagnostic assays and related devices; (iv) evaluation of product deliverability through considerations of packaging, shipping (and/or importing), storage, last mile distribution, and inventory control; (v) establishment of quality assurance and quality control processes for scaled-up manufacture of POC tests; (vi) filing for regulatory approval for the intended use of the technology in a broadly global marketplace; (vii) on-the-ground efforts towards ensuring adoption of the technology by users, clinics, and research laboratories; and (viii) organization of postdelivery support through user training, technical assistance, and maintenance of equipment. Ideally, this chain incorporates end user testing, feedback on performance to the developers, and subsequent cycles of POC test optimization and field testing.

## Barriers to the Successful Development and Distribution of POC Diagnostics in Low Resource Settings

It is common to discuss the “valley of death” analogy as the gap in development between the proof-of-concept demonstration of a new device and its eventual debut in the marketplace. Kumar et al. provide strategies for transitioning from a proof-of-concept to testing in field settings [52], while Chin et al. discuss different cases of commercialization [53]. These, and other reviews on POC diagnostics [54,55], make it clear that there are numerous barriers to the successful implementation of POC diagnostics in resource-limited settings, which can be associated with a particular step along the value chain. Some of the barriers related to international collaborations and technology for global health are discussed below.

### Collaboration between Laboratories across Continents

Workshop attendees universally expressed a desire to set up strong transcontinental collaborations. However, the participants found that transcontinental collaborations can be challenging to establish and maintain due to limited federal funding (e.g., the United States Agency for International Development [USAID]) and other support structures (e.g., the Bill and Melinda Gates Foundation and Grand Challenges Canada) for these kinds of activities. Although successful programs (e.g., the Center for Global Health at Massachusetts General Hospital) and organizations (e.g., PATH and Seeding Labs) exist, extensive institutional mechanisms to enable, foster, and support the growth of these partnerships are needed for change to be possible. While the main goal of most collaborations is to accelerate translation of technologies, attendees felt that developers of technology are often disconnected from the actual needs of the populations they aim to serve. We reached a consensus that these misunderstandings of need are not born from misinformation but rather a lack of information. This gap arises from poor or nonexistent communication among stakeholders in governments, in clinics, and in research laboratories. Failings in communication are not unidirectional; researchers and scientists often do not know the working environment of health care workers in low-resource settings, and, conversely, health care workers in low-resource settings are often not aware of beneficial technological advances. An outcome of this workshop was the understanding that developers should engage end users before tests are designed and research and development (R&D) begins. A challenge with early engagement is obtaining resources to support travel between the developers and end users.

### Critical Gaps in Needs Assessment

Often, there is a lag in time between the actual needs “on the ground” and the recognition of those needs by the global development and research communities. For example, funding mechanisms and calls for proposals in research areas specific to POC diagnostics represent needs at a particular point in time. These areas may be considered the “gold standard” by technology developers even as new challenges and opportunities arise. Although this model has been successfully used to coordinate global efforts, such mechanisms may miss opportunities where new technologies could have an impact. There is a well-recognized shortage of investment in areas of R&D that addresses specific health problems of developing economies [56]. For example, North American and European researchers may consider Rift Valley Fever and plant viruses to be “niche” applications in diagnostics, and yet a strong need exists for diagnostic solutions for these diseases.

Global research priorities are still driven by the “Big Three” (HIV, TB, and malaria), even though a global epidemiological transition has occurred [57]. Noncommunicable diseases now account for almost half of all disability-adjusted life years (DALYs) in low- and middle-income countries [58]. Although this shift is now being realized, and resources reallocated in response, earlier research into low-cost diagnostics for noncommunicable diseases could have helped in the better control of this new burden. For the research community to respond with agility to ever-shifting needs in the field, direct partnerships and assessments are necessary. We would like to see the broader technology development community focus on creating technologies that fit current and future needs rather than forcing ill-suited technologies onto a “hot” topic. Ideally, the R&D cycle begins after establishing a clear understanding of the needs and constraints of the end user and other stakeholders. With the right partners in place to help identify current problems—perhaps using a metric like DALYs directly to influence the funding of research and development programs—it will be easier to convince peers and funders that the research and technology development is worthwhile.

### Flow of Information: Education of Stakeholders, Open Access, and Intellectual Property

#### Educating stakeholders

To realize the full benefits of POC diagnostics, it will be critical to stimulate lasting demand and use by health care workers. Even more important to the successful adoption of POC diagnostics is the need for tests to be as good as, or more reliable than, existing POC tests or tests for diseases that exist only as laboratory tests and do not yet exist as POC tests, and an assurance of continuous quality control processes [59,60]. Previous launches of less-than-successful RDTs demonstrated the need to educate health care workers in proper test usage and inform both health care workers and community members of test reliability and the potential for better clinical outcomes from testing [61,62]. If this information is not made available, the acceptance and utility of these tests can be reduced significantly. End user adoption is critical to success.

#### Open access

Significant disagreement arose surrounding the concept of open access and intellectual property. Currently, there are two strong models emerging for the development of technologies for use in resource-limited settings. One is the open access, do-it-yourself (DIY), or maker movement. This group advocates for complete open access for protocols, designs, and intellectual property so that innovators in the developing world can capitalize freely on knowledge and resources they might not otherwise be able to access. The other group endorses working through for-profit or nonprofit entities that seek sustainable solutions through the enterprise system [63]. The second group maintains that sustainability and scalability can only be achieved by building a business. The first approach reduces the barrier to entry and democratizes science; this approach, however, limits incentives for current market systems to invest in the translation of technologies into marketable products. It also overlooks the financial challenges associated with the development of manufacturing capabilities and the development of distribution streams. Strengths of the second approach include sustainability and a higher quality product with built-in assurances and controls; a disadvantage is slower access to products and the potential for the concentration of knowledge in wealthier countries.

#### Intellectual property

The second approach also includes challenges associated with IP. North American and European academic systems often require that the academic inventors assign ownership of any IP to their academic institution. There are significant costs associated with initiating and completing the patenting process for any new IP. Many universities are unwilling to finance these costs unless a credible opportunity to recover them exists. To realize better returns on their IP portfolios, Western academic institutions are not motivated to defend patents that are not likely to result in high-volume or high-margin products. Thus, this IP often remains unprotected, greatly limiting the ability of the inventor to generate outside funds to commercially develop the technology for wider use. Often, such technologies are demonstrated in pilot projects and then stall due to lack of defendable IP, which further contributes to gaps in the value chain that result in fewer innovations in the marketplace. It may be possible, however, to make this unprotected IP broadly available for uses that may inspire future innovations. Funding mechanisms such as the Bill and Melinda Gates Foundation stipulate that IP emerging from R&D supported by their grants be made available to developing countries at a reasonable cost.

### Barriers in Funding of Collaborations

Many attendees stressed that collaborations between African and Western partners need to happen very early along the value chain, even before the prototyping and design process of new assays. The identification and demonstration of clinical and market needs were frequently stated as the most valuable aspects of these collaborations. Additionally, these relationships enable participants to articulate specific limitations in settings that must be overcome (e.g., cultural or region-specific technological barriers). Another discussion point centered on the lack of innovation infrastructure in many East African countries, which makes it difficult for African inventors to find funding to pursue new research ideas. East African scientists stressed that collaboration within their own institutions across disciplines (e.g., between engineering and biology) remained difficult due to a lack of communication, rendering intercontinental collaborations all but impossible.

A key outcome of the workshop was the identification of a need for grant mechanisms that support initial pilot testing in Africa with African partners. It was noted that many research funding bodies in developed countries do not allow the provision of resources for foreign investigators to be paid fairly for their participation in research projects. For instance, a foreign scientist managing a pilot study of a United States-made device in Africa can often be paid as a consultant. African scientists indicated that it would be preferable to be recognized as a coprincipal investigator and compensated accordingly.

### Biomedical Testing in Low Resource Settings

There is, unfortunately, a long history of Western-based organizations conducting biomedical testing in low-resource settings to skirt regulatory or ethical requirements. A North American or European scientist seeking to test a new technology in Africa should be mindful of this history. Collaborations must use the highest ethical standards to establish research studies. Partnerships realize a greater potential when partners in low-resource settings are viewed as cocreators rather than sources or end users of studies or technologies [64].

## Proposals to Overcome Identified Barriers

After identifying what we consider to be the current main barriers in POC diagnostics, we came to a consensus on several proposed solutions. These proposals do not represent a comprehensive list, but we anticipate that they will function as a starting point for action.

### New Funding Mechanisms

Many of the proposed solutions were related to funding. The level of funding is secondary to the ability of funding mechanisms to work across national and scientific borders. One suggestion is the creation of more calls for proposals that emphasize the participation of multidisciplinary researchers and crosscutting research: for example, grants that focus on translating technologies from the research laboratory to the clinic or grants to study areas like quality control and assurance, which are often orphans in the current funding landscape. The Center for Affordable Medical Technologies’ Innovation Award [65] is an example of what such RFPs might look like.

Scientists and technology developers should become educated in policies and active advocates for changes in global funding paradigms. The negotiations over a proposed Global R&D Treaty at WHO went largely unknown to the scientific communities that would benefit most from such a proposed agreement [66,67]. The scientific community could provide a powerful voice to the debates shaping global research funding; currently, this debate only occurs among policy makers and trade representatives. Innovative funding incentives can bring attention to technologies for neglected and rare diseases.

Also proposed was the establishment of funds for national and international exchange programs for scientists, student researchers, and clinicians. It is immensely useful for biomedical engineers to visit field sites to see processes and challenges in delivering care firsthand. Increasing the numbers of interactions between clinicians and engineers can serve to educate clinicians about what is possible now and what may be possible in the future with ongoing research and collaboration.

### Improved Timing of Collaborations across Borders

There was a strong incentive among workshop attendees to work together and collaborate; however, participants acknowledged the critical disconnects in communication and funding. Without ongoing communication mechanisms—such as through conferences, literature, and real time collaborations—a practical disconnect will remain between the perceived needs of developers and end users. These mismatches will continue to grow in the absence of collaborations.

It was also noted that developers of technologies and POC diagnostics should clearly communicate timelines to potential end users. When this timeline—for the development pipeline and for product launch—is not clear, end users often grow wary of hearing about “the next new thing” that is promised to save lives but never arrives. When obstacles in the value chain are acknowledged before product development, some of these misperceptions can be avoided. Western scientists may be rewarded by granting mechanisms for overly optimistic projections of success in terms of time and impact. There are real disadvantages in terms of buy-in from African partners when these projections are unmet. It is the responsibility of technology developers to be pragmatic and set realistic expectations.

There are several ways for funders, governments, and policy makers to promote active collaborations between individuals and institutions. First, they can facilitate contact between groups. It is often difficult for technology developers and clinical practitioners to find collaborative partners with mutual interests and complementary skills. Our workshop provided a venue for such connections to begin to percolate; however, only with repeated interactions that build familiarity and trust between participants can strong and mutually-supportive collaborations be forged from trust and familiarity. Support for other international conferences is one way to foster new partnerships. Other mechanisms include using social media and the Internet to connect people with similar interests and complementary skill sets. Establishment of a virtual community of technology developers, scientists, and clinicians similar to the Global Health Delivery Online (www.ghdonline.org) community could provide a secure forum for the active exchange of ideas for those without the resources for travel. GHDonline is a free, subscription-based website that requires users to log into a social media environment. This semiprivate, moderated forum has been very useful to clinicians all over the world. We believe that new collaborations could nucleate from such an environment. A moderated online forum could also serve as a venue for the training, technology assessment, and dissemination of results. Moderation requires funding; we feel that an unmoderated site would be unsuccessful. Second, the establishment of proper protocols for research is confusing and difficult to navigate when scientific teams consist of individuals from different countries. Funding agencies could support and promote country specific mentoring services to shepherd investigators through the process. Stakeholders in technology development and in patient care could greatly benefit from such mentoring.

### Education of Stakeholders

There are often barriers to the clinical adoption of POC tests due to the lack of information or misinformation about the tests. Users will not transition to a new test simply because it is touted as better than the standard of care; they must be convinced with data and trained by knowledgeable users. In the best case, these knowledgeable users are their peers. Trust between trainers and technology-adopters is paramount when patient outcomes are at stake. The following mechanisms have the potential to mainstream simple tests into routine medical practice: (i) develop and update health curricula to reflect advances in POC diagnostics; (ii) provide refresher workshops to educate health care workers on developments in POC diagnostics; (iii) disseminate updates of information or progress using mass media; (iv) establish dedicated online resources for the use and interpretation of POC diagnostics.

Barriers can also arise from misunderstandings. In some cases, there is a misperception that POC tests will completely replace clinical laboratories and extract jobs and financial support from hospitals. POC tests will ideally augment medical systems found within clinical laboratories rather than to replace clinical laboratories altogether. Working with clinical laboratories during development and communicating objectives can avoid misunderstandings from becoming obstacles. The challenges associated with distributing POC tests to end users can drive the development of business models that create new jobs, such as health care workers that buy the tests, transport them to test sites, and perform the assays.

Another important avenue for education is the scientific literature. New technologies and discoveries are often published behind prohibitively expensive paywalls that prevent end users in both developed and developing countries from accessing valuable knowledge about effectiveness and validation. Open-access publishing (e.g., the Public Library of Science) and open access archives (e.g., ArXiv and bioRxiv) provide two alternatives that can reduce barriers to access and enable the education of stakeholders. Open access to high quality scientific literature will ultimately promote the adoption of the best technologies available to solve appropriate problems.

### Re-evaluation of the Current Paradigms in POC Technology Promotion

When the Bill and Melinda Gates Foundation and other agencies set forth a list of priorities for POC testing in low-resource settings approximately one decade ago, a number of assumptions were made regarding the forward progress of the field. We feel that a reexamination of those assumptions is warranted in the context of what has been learned in the past ten years. For example, one of the original papers suggests: “… when a new test is introduced, it will initially be available to the providers with the most sophisticated infrastructure, followed by those with progressively worse infrastructure” [68]. It is unclear whether such an implementation model has been successful.

Further, the cost of a POC test—including necessary labor, transportation, storage, and quality control—in many cases must be very low to offer a true advantage. In some cases, the decrease in cost and increase in functionality of associated technologies, like mobile devices, may shift the balance back toward a model that approximates the function of a centralized laboratory. A model consisting of regional centers with testing equipment can be more expensive and have a slower turn-around time than centralized testing performed at a single national center but combined with mobile reporting systems.

The assumption that the lowest cost test will be the best test for low resource settings was challenged at the workshop. Many participants noted pushback from clinicians and patients when an inexpensive test was perceived as shoddy or substandard. The presence of one poorly executed test in the marketplace can negatively impact end user perceptions of POC tests. Quality control and test validation has emerged as an area of increased concern over the last decade. It has been well documented that pharmaceuticals are substandard or counterfeited at alarming rates in both low- and high-resource settings [69]. It is a lesser-acknowledged problem that POC diagnostics are also subject to poor regulatory oversight and counterfeiting activity [70]. When quality control is poor and tests do not result in better patient care, those tests will be rejected by patients and clinicians. The group identified a need for POC diagnostics with built-in quality control and quality assurance measures. Incorporating appropriate innovations, such as temperature-monitoring labels, serializing tests, mobile phone–based product verification, and tracking expiration dates were described. Mechanisms for postmarket surveillance are challenging in many settings, but this need should be addressed by both manufacturers and regulatory agencies.

### Promotion of African Research Laboratories

We believe that strengthening the ability of laboratories in low-resource settings to perform innovative research in the area of POC diagnostics is an investment that could have the most transformative impact on the field. African scientists are uniquely positioned to address all of the links along the local value chain. There are several nascent groups with the potential to galvanize the scientific community interested in diagnostics development in Africa. Examples include the African Society for Laboratory Medicine (www.aslm.org) and African Network for Drugs and Diagnostics Innovation (http://www.andi-africa.org ). Analogies were drawn to the recent surge in innovation surrounding mobile technologies in Africa. The “Silicon Savannah” concept has largely been applied to information technologies and not to biomedicine [71]. These organizations should aim to continue to promote and strengthen the concept of African innovation in translational biomedicine.

It was also noted that public universities in Africa do not yet have the same culture of interdisciplinary research as in the Western world. Efforts to promote research that crosses over traditional academic “silos” is necessary to generate crossdisciplinary research and realize new POC technologies. Funding agencies and governments can have an impact on this kind of collaboration within African institutions. Many North American and European universities have adopted more crosscutting research models in the biological sciences. The transition has taken years, due to the lack of clear rewards for participating in collaborative research (as opposed to working alone). In the US, the National Institutes of Health has focused on translational research through the creation of a new center in 2011 [72], and several private funders have announced requests for proposals that require a collaborative approach. These changes, and others, have begun to drive research in more multidisciplinary directions. Strengthening of the regulatory infrastructure in African countries was cited as another investment that could greatly change the impact of African innovation. Clear mechanisms for getting legal approval and for postmarket surveillance are needed if devices are to make it to market quickly and patient safety is to be maintained.

### Sustainability

Overarching all of these recommendations is the need for sustainability. It is not enough to bring a new test to the marketplace and have it be adopted. Supply chains must be maintained, procurement must be simplified, and repairs or changes in protocols must be easy to communicate to the end user. All of these components require systems-level thinking on the part of the technology developers and distributors. Practical issues of sustainability should be discussed early and revisited often. Further, the value of POC diagnostics to overall health care must be made clear to funders and governments in quantitative terms. Use of POC diagnostics should be included in epidemiological and economic modeling studies in order to attach a value to the assay or the technology. When the use of a POC diagnostic improves health care outcomes and reduces the time to initiate as well as the cost of treatment, it becomes easier to lobby for the allocation of future expenditures to be directed towards the deployment of specific POC diagnostics.

Several attendees suggested the establishment of mechanisms for the sustainable production of the solution (i.e., the POC diagnostic) in the country or region of use. These mechanisms include protocols for transferring technologies from a developed country to a developing country. Also important is the stimulation of market forces to drive the development and implementation of these new technologies. Sustainability depends on building capacity in Africa for interdisciplinary training and research. Ideally, technologies will arise within Africa and remove the current challenges associated with the transfer of technology between developed and developing countries. Student and scientist exchanges will also support sustainability by enabling African scientists to gain technical skills and interdisciplinary training and teaching North American and European scientists how to innovate in low-resource settings and to assess specific needs.

## Summary

A major goal of the 1st International Point-of-Care Diagnostic Workshop in Nairobi, Kenya was to provide a forum for open dialog concerning current challenges in, and potential solutions for, the development of the next generation of POC diagnostics. The focus was not solely on descriptions of new technologies in development but also included demonstrations of their use in a field setting. The resulting conversations identified a number of obstacles to the successful translation of prototypes into field-deployable tools. These obstacles superseded those typically encountered in research; changes must be implemented at the institutional and governmental level to enable equitable collaborations between Western and African partners, and proper funding mechanisms must be established to support these collaborations. Additionally, this workshop showcased emerging technologies for POC tests and fostered new partnerships between technology developers and African research laboratories. Equitable partnerships are critical for the successful implementation of new POC technology. The attendees agreed that the most effective methods to effect change require improved communication of needs, ideas and abilities, and a conduit for the sharing of experiences and information. We plan to implement many of the changes that are suggested here in our own research programs and to use future conferences and workshops to guide the development of both technologies and partnerships. Our successes and failures will serve as models for those scientists striving to develop technological and biomedical solutions to similar problems in global health.

## Supporting Information

S1 Text The conference program containing full description of the devices, list of conference presenters, attendees, and abstracts of the presentations. (PDF)
