# Correction: Enabling the Development and Deployment of Next Generation Point-of-Care Diagnostics

**DOI:** 10.1371/journal.pntd.0003857

**Published:** 2015-06-15

**Authors:** The PLOS Neglected Tropical Diseases Staff

On page 2 of the PDF, the second link in the last sentence of the first paragraph of the Introduction is incorrect. Please see the correct link here: https://www.youtube.com/watch?v=cYTs3FoOOo4.
