# Supplementary material for: Enabling the Development and Deployment of Next Generation Point-of-Care Diagnostics
Source: PLoS Negl Trop Dis. 2015 May 14;9(5):e0003676. doi: 10.1371/journal.pntd.0003676 (PMC4431858; doi:10.1371/journal.pntd.0003676)
Supplement: S1 Text — (PDF) [file pntd.0003676.s001.pdf]

Bringing Diagnostic Prototypes

TO THE POINT OF CARE

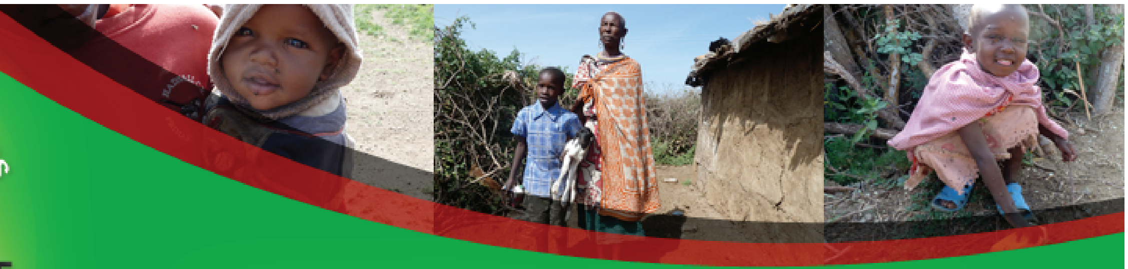

1ST ANNUAL

HANDS-ON WORKSHOP IN POINT-OF-CARE DIAGNOSTIC

Institute of Primate Research
National Museums of Kenya
Nairobi, Kenya

June 25th - 29th, 2012

Conference Program

Institute of Primate Research

UNIVERSITY OF ALBERTA
EDMONTON·ALBERTA·CANADA

Alberta Glycomics Centre

SENTINEL
BIOACTIVE PAPER NETWORK

Grand Challenges Canada
Grands Défis Canada

Welcome to NAIROBI, KENYA, AFRICA

Table of Contents

Schedule ……………………………………………………………………………………... 2-6

Presenter Abstracts.………………………………………………………………………… 7-33

List of Posters ……………………………………………………………….………...... 34-36

Poster Abstracts ………………………………………………………….………… 37-74

List of Participants …………………………………………………………………....….75-77

This conference was organized by Dr. Ratmir Derda and Karen McKinley (University of Alberta) and Organizing Committee of the Institute of Primate Research (Nairobi, Kenya): Dr Tom Kariuki, Dr Evans Taracha, Dr John Kagira, Dr Atunga Nyachieo and Ms Gladys Gichuhi

This conference was made possible by the financial support from the following organizations:

The Alberta Glycomics Centre is a discovery, research, and technology group dedicated to basic research on carbohydrates that will advance understanding of their role in life processes. The Alberta Glycomics Centre is an initiative of the Alberta Ingenuity Fund. The Alberta Ingenuity Centre program provides support for outstanding researchers from Alberta post-secondary institutions that work in areas of strategic importance to the province, and will contribute to the diversification and growth of Alberta's economy and quality of life through the technologies and jobs created.

Alberta Glycomics Centre

Student and speaker travel awards were supported by Faculty of Science and Department of Chemistry at the University of Alberta, Edmonton.

UNIVERSITY OF ALBERTA

Grand Challenges Canada (GCC) aims to develop a consortium of world-leading Canadian and International scientists, research organizations, and leaders from the business sector to develop breakthrough solutions to global challenges and ensure that these solutions are available to those who need them the most. The mission of GCC is to identify global grand challenges, fund a global community of researchers and related institutions on a competitive basis to address them, and support the implementation/commercialization of the solutions that emerge

Grand Challenges Canada™
Grands Défis Canada
BOLD IDEAS FOR HUMANITY.™
DES IDÉES AUDACIEUSES POUR L'HUMANITÉ.

The cornerstone of the SENTINEL vision is bioactive paper that will detect, capture and deactivate water and airborne pathogens. Current events including SARS, water quality problems, incidents of tainted beef and international terrorism underscore our vulnerability to biological hazards.

SENTINEL BIOACTIVE PAPER NETWORK

The session about Contagious Bovine Pleuropneumonia is funded by the German Federal Ministry for Economic Cooperation and Development (BMZ)..

BMZ Federal Ministry for Economic Cooperation and Development

**Sunday, June 24, 2012**		
	**Conference Registration**	**Karen Country House Inn Hotel, Or Karen Embers Hotel**
**Monday, June 25, 2012, Location: NMK**		
7:00 - 8:00 am	**Breakfast**	**Respective Hotels**
8:00 am	**Travel to National Museums of Kenya (NMK)**	
**SESSION 1 NMK Main Auditorium**		Chair: Ratmir Derda, Evans Taracha
9:00 am	**Opening remarks**<br>**Dr. Mark Bor -** Permanent Secretary, Ministry of Public Health and Sanitation	
9:30 am	**Thomas Kariuki – Institute of Primate Research**<br>*Overview of Research on Diagnostics at IPR*	
10:00 am	**Coffee Break**	**NMK Courtyard**
10:30 am	**Joseph Ndung'u - Head, HAT & OND, Foundation for Innovative New Diagnostics (FIND)**<br>*Advances in development of point of care diagnostics for sleeping sickness*	
11:05 am	**Julianne Gibbs-Davis – University of Alberta**<br>*Optimizing DNA Detection using Amplification by Destabilization*	
11:40 am	**Paul Yager - University of Washington**<br>*Development of Point-of-Care Medical Assays in Paper*	
12:15 pm	**Lunch**	**NMK Poster Hall**
**SESSION 2 NMK Main Auditorium**		Chair: Charles Mace
1:30 pm	**Marya Lieberman - University of Notre Dame**<br>*Detection of counterfeit pharmaceuticals with paper analytical devices*	
2:05 pm	**Michael Serpe – University of Alberta**<br>*Biosensing with Polymer-Based Color Tunable Materials*	
2:40 pm	**Andres Martinez – Cal Poly**<br>*Fully-enclosed Paper-based Fluidic Devices*	
3:15 pm	**Coffee Break**	**NMK Courtyard**
3:45 pm	**Emanuel Carrilho – University of Sao Paulo**<br>*Wax Printed Microfluidic Paper-based analytical Devices: From Conception to Application*	
4:20 pm	**Ratmir Derda – University of Alberta**<br>*Diagnostic for TB-antibodies based on phage and portable bacteria cultures*	
4:55 -5:30 pm	**Marc Dusseiller - Dusjags labs, hackteria.org**<br>*Biohacking - Democratization through Demystification of Bio- and Nanotechnology*	
6:30 pm	**Dinner**	**Location TBA**

Tuesday, June 26, 2012. Experimental Session. Location: IPR<br>International Facilitators: Dr Atunga Nyachieo and Dr John Kagira		
7:00 - 8:00 am	**Breakfast**	**Respective Hotels**
8:00 am	**Travel to Institute of Primate Research (IPR)**	
9:00 am	**Demo Set-up**	
9:30 am - 1:00 pm	**Diagnostic Demonstrations**<br>**Demo Presenters:**<br>Joseph Ndung'u<br>Julianne Gibbs-Davis<br>Paul Yager<br>Marya Lieberman<br>Michael Serpe<br>Andres Martinez<br>Emanuel Carrilho<br>Ratmir Derda<br>Marc Dusseiller<br>Poster presenters with demos (Posters 1-30)	
1:00 pm	**Lunch**	**IPR courtyard**
2:00 pm	**Demonstration Discussions**	
3:00 pm	**Travel to Point-of-Care clinic near Ngong**<br>Group A	
3:30 - 5:00 pm	**Tour Point-of-Care clinic**<br>Group A	
6:00 pm	**Dinner**	**Location TBA**

Wednesday, June 27, 2012, LocationL NMK		
7:00 - 8:00 am	**Breakfast**	**Respective Hotels**
**SESSION 3 NMK Main Auditorium**		Chair: Julianne Gibbs-Davis
8:00 am	**Travel to National Museums of Kenya**	
9:00 am	**Catherine Klapperich – Boston University**<br>*Low Cost Diagnostics for Monitoring Drug Regimen Adherence*	
9:35 am	**Doug Weibel – University of Wisconsin**<br>*BacChip: Portable Hands-free system for bacterial identification and treatment*	
10:10 am	**Coffee Break**	**NMK Courtyard**
10:25 am	**Jonas Tegenfeldt – Lund University**<br>*Pathogen enrichment using deformability and morphology based sorting*	
11:00 am	**Hywel Morgan – University of Southampton**<br>*The Point of Care Complete Blood Count*	
11:35 am	**Coffee Break**	**NMK Courtyard**
11:50 am	**Michelle Khine – University of California**<br>*Shrink-Induced Manufacturing Process for Low Cost Diagnostics (SIMPL-CD)*	
12:25 pm	**William Shu – Heriot-Watt University**<br>*Manually actuated microfluidic device for blood plasma separation*	
1:00 pm	**Lunch**	**NMK Poster Hall**
**SESSION 4 NMK Main Auditorium**		Chair: Michael Serpe
2:00 pm	**Jonathan Cooper – University of Glasgow**<br>*TBA*	
2:35 pm	**Charles Mace – Diagnostics For All (DFA)**<br>*Low-Cost Diagnostics to improve small farmer income in resource-constrained settings*	
3:10 pm	**Ashok A. Kumar (A.J.) – Harvard University**<br>*Diagnostics by Density*	
3:45 pm	**Coffee Break & Poster set-up**	**NMK Poster Hall**
4:15 - 5:30 pm	**Poster Session**	**NMK Poster Hall**
6:30 pm	**Dinner**	**Location TBA**

Thursday, June 28, 2012. Experimental Session. Location: IPR<br>International Facilitators: Dr Atunga Nyachieo and Dr John Kagira		
7:00 - 8:00 am	**Breakfast**	**Respective Hotels**
8:00 am	**Travel to Institute of Primate Research**	
9:00 am	**Demo Set-up**	
9:30 am - 1:00 pm	**Diagnostic Demonstrations**<br>**Demo presenters:**<br>Catherine Klapperich<br>Dough Weibel<br>Jonas Tegenfeldt<br>Hywel Morgan<br>Michelle Khine<br>William Shu<br>Jonathan Cooper<br>Charles Mace<br>AJ Kumar<br>Poster presenters with demos (Posters 30-60)	
1:00 pm	**Lunch**	**IPR Courtyard**
2:00 - 3:00 pm	**Demonstration Discussions**<br>**Location: IPR Seminar Hall**	
3:00 pm	**Travel to Point-of-Care clinic near Ngong**<br>Group B	
3:30 – 5:00 pm	**Tour Point-of Care clinic**<br>Group B	
6:00 pm	**Dinner**	**Location TBA**

Friday, June 29, 2012, Location: NMK		
7:00 - 8:00 am	**Breakfast**	**Respective Hotels**
8:00 am	**Travel to National Museums of Kenya**	
**SESSION 5 NMK Main Auditorium**		Chair: Martin Thuo
9:00 am	**Reginald Beer - Lawrence Livermore National Laboratory**<br>*Cooperative Threat Reduction for the 21st Century*	
9:35 am	**Nana Yaa Boadu – University of Alberta**<br>*Rapid malaria diagnosis in Ghana: implementing policies, and navigating technology at the point of care*	
10:10 am	**Daniel Maina – Aga Khan University Hospital**<br>*Challenges in establishing Point of Care Testing in a developing country; The Aga Khan University Hospital experience*	
10:45 am	**Coffee Break**	**NMK Courtyard**
11:00 am	**Panel Discussion**	
12:00 pm	**Lunch**	**NMK Poster Hall**
**SESSION 6 NMK Main Auditorium**		Chairs: A.J. Kumar and Evans Taracha
1:00 – 3:00 pm	**Rapid-fire talks – 20mins each**<br>**Martin Mwangi Thuo -** Harvard University<br>*Low-cost Microfluidic Devices derived from Ultra-hydrophobic paper*<br>**Claire Mugasa** – Makerere University, *Comparison of nucleic acid sequence-based amplification and LAMP for diagnosis of sleeping sickness*<br>**Christopher Kariuki -** Institute of Primate Research, *Development of a nanobody® based diagnostic tool for Trypanosoma brucei rhodesiense*<br>**Lucy Ochola** – Institute of Primate Research, *Malaria diagnosis: need to track the changing face of malaria transmission*<br>**Imna Malele** – Jomo Kenyatta University of Agriculture and Technology, *Importance of rapid and sensitive diagnostic in accurate estimation of Human African Trypanosomiasis magnitude in endemic areas in Tanzania*<br>**Elizabeth Ochola** – KEMRI-KSM, *A Comparison of Diagnostic Methods against PCR for the detection of* S. mansoni *among school Children in Western Kenya*	
2:40 pm	**Coffee Break & Poster Set-up**	**NMK Poster Hall**
3:00 - 5:00 pm	**Contagious Bovine Pleuropneumonia Session**<br>**Discussion Leader: Joerg Jores**	**NMK Main Hall**
3:00 - 5:00 pm	**Additional Poster viewing (same posters as Wednesday session).**	**NMK Poster Hall**
5:00 - 5:30 pm	**Closing Remarks**<br>**Dr Hezekiah Chepkwony,** Director National Quality Control Laboratory, Kenya	**NMK Main Hall**
6:30 pm	**Dinner**	**Location TBA**

Dr. Joseph Ndung'u
Head, HAT & OND, Foundation for Innovative New Diagnostics (FIND)

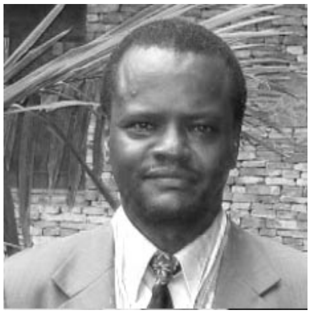

Dr. Ndung'u joined FIND in 2006 from the Kenya Agricultural Research Institute (KARI), where he was Chief Research Officer. Before moving to KARI, he was the Director of the Kenya Trypanosomiasis Research Institute (KETRI), which he joined in 1985. Dr. Ndung'u's area of specialization is in immunopathology. He has published widely on human and animal health in local and international journals, and has contributed several book chapters in his field. Dr. Ndung'u has been a visiting professor at the University of Wisconsin in Madison (USA) and the University of Glasgow (UK), and is a member of a number of international consortia and committees.
http://www.finddiagnostics.org/about/who_we_are/our_people/team/ndungu.html

Abstract:

ADVANCES IN DEVELOPMENT OF POINT OF CARE DIAGNOSTICS FOR SLEEPING SICKNESS

Joseph M. Ndung'u

Foundation for Innovative New Diagnostics (FIND), 16 Avenue de Budé, 1202 Geneva, Switzerland
Email: josepg.ndungu@finddiagnostics.org

Human African trypanosomiasis (HAT) is a disease of poor rural communities caused by extracellular protozoan parasites of the genus Trypanosoma. In early or Stage 1 infection when parasites are in blood and the lymphatic system, treatment is relatively safe and cheap. During this time however, clinical signs are not suggestive of HAT, and diagnostic tests have problems of sensitivity and specificity. Many cases therefore remain undetected and parasites invade the brain, resulting in late or Stage 2 disease, and symptoms associated with HAT are observed. Treatment of Stage 2 HAT is lengthy and expensive, and the drugs used can cause potentially fatal side effects. Unfortunately, tests to determine the stage of disease are non-specific and insensitive. Early and accurate diagnosis and staging of HAT would ensure safe treatment, reduced transmission and accelerated control of the disease. Major steps in development of novel tests for diagnosis of HAT, and for determining the stage of disease have been made recently. A simple rapid test for screening populations is undergoing evaluation. Individuals found positive with this test will be confirmed by demonstration of parasites using an LED fluorescence microscope, or by a simple molecular method called loop-mediated isothermal amplification (LAMP) of DNA. Biomarkers that accurately distinguish Stage 1 and 2 disease have also been identified in spinal fluid of HAT patients. Completion of development of these new tests and their introduction for diagnosis of HAT is likely to accelerate control and the eventual elimination of the disease.

Dr. Julianne M. Gibbs-Davis
University of Alberta, Edmonton, AB; Canada

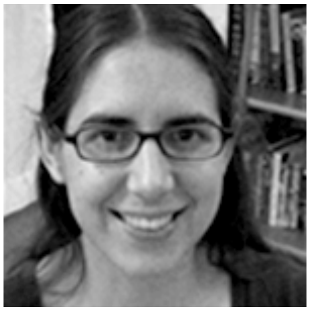

Juli grew up in Northern Arizona and received her B.A. in chemistry from Arizona State University. She pursued her PhD with SonBinh Nguyen in the chemistry department at Northwestern University on the molecular recognition properties of polymer-DNA hybrid materials. As a Dreyfus Postdoctoral Fellow in Environmental Chemistry she worked with Franz Geiger and Karl Scheidt at Northwestern on combining surface chemistry and nonlinear optical spectroscopy to tackle questions about environmental interfaces related to pollutant transport and atmospheric aerosol oxidation. Her group is interested in how molecular recognition occurs in confined environments like materials interfaces. Using this information, they are developing strategies for tuning molecular recognition to create new functional materials with applications in diagnostics and therapeutics.

Abstract:

OPTIMIZING DNA DETECTION USING AMPLIFICATION BY DESTABILIZATION

Juli Gibbs-Davis

Department of Chemistry, University of Alberta, Edmonton, Alberta, Canada
Email: gibbsdavis@ualberta.ca

We have developed a system of DNA amplification that allows for turnover numbers of greater than 1 million to be obtained at a single temperature, often close to room temperature. This isothermal amplification method has great potential in infectious disease detection particularly of drug resistant strains, which can be discriminated by single mutations present in their genome. The novelty behind our system is the incorporation of a destabilizing chemical modification into the DNA, which facilitates dissociation of the newly formed complementary sequences from the original target templates, without temperature cycling.[1] For this system to be applied to point-of-care diagnostics, three requirements must be met: first, it has to be capable of detecting small amounts of target DNA with minimal sample purification; second, the optimal temperature for the reaction needs to be tunable so that it can be used in the field or in other resource-limited settings, and third the read-out methods need to be simple and affordable. The first requirement was met by performing a serial ligation method that allows us to detect high fM concentrations of DNA with minimal system optimization. The second criteria was achieved by using different combinations of destabilizing groups. Finally, the read-out method was simplified from a two-step process consisting of target separation with polyarcrylamide gel electrophoresis and detection with a computer-controlled imager, to a one-step method using a molecular beacon, an LED flashlight, and a simple excitation filter.

1. Kausar et al. Angew Chem. Int. Ed 2011, 50, 8922

Dr. Paul Yager
University of Washington, WA, USA

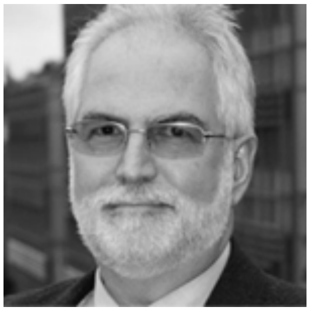

Dr. Yager received his A.B. in Biochemistry from Princeton (1975), and a Ph.D. in Chemistry from the University of Oregon (1980). He was an NRC Fellow at the Naval Research Laboratory in DC from 1980 to 1982, joining the NRL staff in 1982. He joined the Department of Bioengineering at the University of Washington in 1987, and became Chair in 2008.

For the last two decades, work in the lab has focused on development of microfluidic devices for monitoring of medically-significant analytes. The primary goal of current work is increasing access to healthcare through decentralization of biomedical diagnostic testing. A grant from the Bill & Melinda Gates Foundation developed a low-cost point-of-care platform for diagnosing diseases in the developing world, now being developed by Micronics, Inc. Since 2008, the lab has focused on instrument-free diagnostics based on 2-dimensional paper networks. Specifics can be found at http://faculty.washington.edu/yagerp/.

Abstract:

DEVELOPMENT OF PONT-OF-CARE MEDICAL ASSAYS IN PAPER

Paul Yager, Elain Fu, Barry Lutz

Department of Bioengineering, Box 355061, University of Washington, Seattle, WA 98195, USA
Email: yagerp@uw.edu

Two-dimensional paper networks (2DPNs) allow complex chemical processing in a very low-cost format. We have, for the last 4 years, been learning how to translate what we have learned about point-of-care diagnostic technologies in conventional microfluidics into the language of porous media. The wicking of fluids in porous materials (like paper, nitrocellulose membranes, etc.) allows us to discard pumps, which allows great savings in complexity, and the potential to perform complex tests without any permanent instruments. However, there are many physical and chemical differences between open ducts and porous media. We have put a good deal of effort into understanding the performance and design rules of simple paper systems. Currently, the primary applications for this technology in our lab are highly-sensitive multiplexed immunoassays and multiplexed isothermal nucleic acid amplification assays. All assays are designed with visible optical readout that can be captured and quantified using camera-equipped cellular phones.

Dr. Marya Lieberman
University of Notre Dame, IN, USA

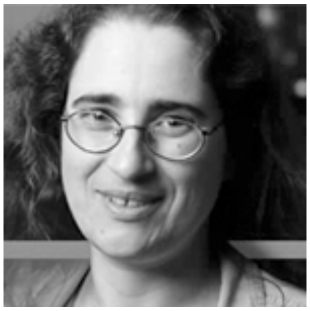

Marya Lieberman is an associate professor in the Department of Chemistry and Biochemistry at the University of Notre Dame in South Bend, Indiana. Her research is in the area of surface chemistry, self-assembly of two- and three-dimensional structures, and molecular electronics. A new project focuses on detection of counterfeit drugs with paper-based sensors and cell phone imaging; this technology is currently being field tested in Eldoret, Kenya.

Abstract:

DETECTION OF COUNTERFEIT PHARMACEUTICALS WITH PAPER ANALYTICAL DEVICES

Marya Lieberman

Department of Chemistry and Biochemistry, University of Notre Dame, Notre Dame, IN 46556, USA
Email: mlieberm@nd.edu

We are developing inexpensive analytical devices to detect fake pharmaceuticals. The devices are paper-based, contain all necessary reagents, do not require power, and can be read by taking a picture with a cell phone and sending it to a web site. These fast screening tests can single out suspicious drugs for confirmatory testing and greatly reduce the cost of monitoring critical pharmaceuticals. The fabrication method involves thermal wax printing and deposition of reagents using a micropipette or a device called a "frog". Image analysis methods will be discussed & shared.

Dr. Michael Serpe
University of Alberta, Edmonton, AB; Canada

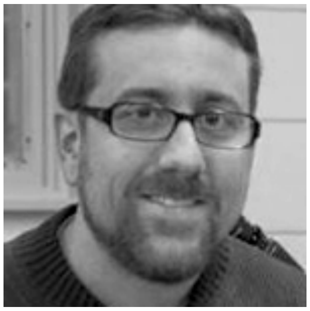

Dr. Michael J. Serpe received his Ph.D. in Analytical Chemistry from the Georgia Institute of Technology in 2004. After a brief stint in industry, Dr. Serpe began his postdoctoral position at Duke University in 2006. He has been an Assistant Professor of Analytical Chemistry at the University of Alberta since 2009. His group is developing polymer-based systems to solve problems in both health and the environment.

Abstract:

BIOSENSING WITH POLYMER-BASED COLOR TUNABLE MATERIALS

Michael Serpe

Department of Chemistry, University of Alberta, Edmonton, AB, T6G 2G2, Canada
Email: michael.serpe@ualberta.ca

Color tunable materials have been generated by depositing a poly (N-isopropylacrylamide) microgel layer between two, thin Au layers, all on a glass support. Color results from constructive/destructive interference of light resonating in the polymer-based cavity. The observed color is directly dependent on the distance between the two metal layers. We have been exploiting this phenomenon for a variety of sensing applications. Most recently, we showed that the material's color depends on the amount of glucose present in a solution; at low concentration, the device is green, while it appears red at high glucose concentration. This presentation will highlight our most recent efforts related to biosensing.

Dr. Andres Martinez
Cal Poly, CA, USA

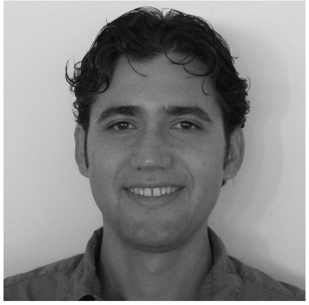

Andres W. Martinez is an Assistant Professor in the Department of Chemistry and Biochemistry at California Polytechnic State University, San Luis Obispo. He was born in California, raised in Bolivia, and completed B.S. in chemistry at Stanford University and his Ph.D. in chemistry at Harvard University with George Whitesides. His research group is currently focused on expanding the capabilities of paper-based fluidic devices for applications in point-of-care diagnostics

Abstract:

FULLY-ENCLOSED PAPER-BASED FLUIDIC DEVICES

Andres W. Martinez

California Polytechnic State University - San Luis Obispo
Email: awmartin@calpoly.edu

Paper-based fluidic devices constitute a promising platform for point-of-care diagnostic devices. A feature common to all these devices is that the top and bottom faces of the channels in the devices remain open and exposed to the environment, which can lead to contamination and loss of samples due to evaporation. This talk will describe recent developments in the fabrication of fully-enclosed devices where the top and bottom faces of the channels are sealed with a layer of toner applied using a conventional laser printer.

Our work is focused on expanding the capabilities of point-of-care diagnostic devices made out of patterned paper by developing simple methods for enclosing the devices, for controlling fluid movement within the devices and for fabricating layered three-dimensional devices. We will demonstrate the fabrication and expanded capabilities of these devices using simple diagnostic assays for glucose and alkaline phosphatase

Dr. Emanuel Carrilho
University of Sao Paulo, Brazil

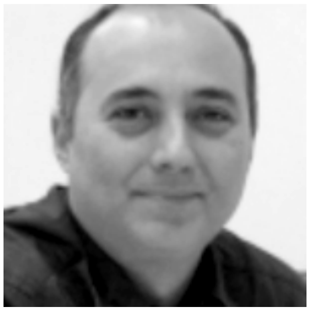

Dr. Carrilho obtained his B.Sc. in Chemistry (1987) and M.Sc. in Analytical Chemistry (1990) from the University of São Paulo (USP) at São Carlos, Brazil. From 1993 to 1997 he obtained his Ph.D. at the Northeastern University under the mentoring of Professor Barry L. Karger, from the Barnett Institute, in Boston, MA. He joined the faculty of the analytical chemistry program of the Institute of Chemistry at São Carlos, USP in 1998, where in 2004, became Associate Professor. During 2007-2009 he was a visiting professor at Harvard University in Professor George M. Whitesides group.

"Our lab makes selective assays to test for a variety of analytes, from food safety to health monitoring. Our platform of choice is microfluidic paper-based analytical devices (µPADs) made with wax printer, in both lateral flow and 96-well plate format."

Abstract:

WAX PRINTED MICROFLUIDIC PAPER-BASED ANALYTICAL DEVICES: FROM CONCEPTION TO APPLICATION

Emanuel Carrilho,[1,2] Maribel E. Funes-Huacca, Thiago Mazzu, Rubiane A. Borba

[1]*Institute de Química de São Carlos, Universidade de São Paulo, São Carlos – SP, Brazil*
[2]*Instituto Nacional de Ciência e Tecnologia de Bionalítica – INCTBio, Campinas, SP, Brazil*
Email: emanuel@iqsc.usp.br

Microfluidic paper-based analytical devices (µPADs) can be considered as the latest, and maybe the most fast-growing technology for the detection of analytes and pathogens at extremely low cost. We present three examples of applications presenting variety of *i*) designs (lateral flow and microzone plates), *ii*) chemistry of detection (nanoparticles and dye chemistry), as well as *iii*) type of analytes (organic compounds, small inorganic anion, and bacteria) and *iv*) assays (colorimetric, enzymatic, and immunoassays). Microfluidic paper-based analytical devices are a promising technology to develop simple, low-cost, portable, and disposable diagnostic platform for resource-limited settings. This technology is based in colorimetric bioassays for direct detection of contaminants, metabolites, drugs, diagnostics of disease, etc. Then, the analysis of image is evaluated directly from scanners, or even from cell-phone cameras for telemedicine applications. We report examples of the fabrication of µPADs in cellulose membrane of Whatman #1 chromatography paper by wax printing, in which we apply for *i*) detection of adulteration of protein content with melamine and *ii*) analysis of nitrite in saliva for periodontitis, using a lateral flow µPAD, and *iii*) immunoenzymatic assays for *Toxoplasma gondii* detection in pregnant women, using a 96-microzone paper plate.

Dr. Ratmir Derda
University of Alberta, Edmonton, AB; Canada

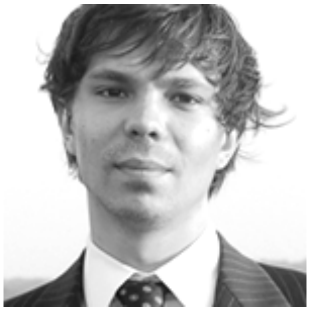

Ratmir Derda is an Assistant Professor in Chemistry at the University of Alberta, Principal Investigator at the Alberta Glycomics Centre and researcher at the SENTINEL Bioactive Paper Network. He received his B.Sci. in Physics from Moscow Institute of Physics and Technology in 2001 and Ph.D. in Chemistry from the University of Wisconsin-Madison in 2008, under the supervision of Laura L. Kiessling. From 2008 to 2011, he was a postdoctoral researcher at Harvard University and the Wyss Institute for Biologically Inspired Engineering under the supervision of George M. Whitesides and Donald E. Ingber. His notable awards include Canadian Rising Star in Global Health award from Grand Challenges Canada (2011), National Academies Keck Futures Initiative Award in Synthetic Biology (2010), ACS Excellence in Graduate Polymer Science Research (2007) and Gold Medal at the XXIX International Chemistry Olympiad (1997)

Abstract:

DIAGNOSTIC FOR TB-ANTIBODIES BASED ON PHAGE AND PORTABLE BACTERIA CULTURES

Ratmir Derda

Department of Chemistry and Alberta Innovates Centre for Carbohydrate Science, University of Alberta, Edmonton, AB T6G 2G2, Canada
Email: ratmir.derda@ualberta.ca

The Derda Lab is interested in simple solution for biochemical assays and for culture of cells and bacteria in limited-resource environment. We also promote the concept of “point-of-care production” for point-of-care diagnostic devices. In this presentation we will demonstrate a functional, portable device for the growth of bacteria that can be created using simple materials. These devices are comprised of packing tape, sheets of paper patterned by hydrophobic printer ink, and a polydimethyl siloxane (PDMS) membrane, which is selectively permeable to oxygen but non-permeable to water. The devices supply bacteria with oxygen and prevent the evaporation of media for a period over 48 hours. The division time of bacteria in this culture are similar to the rates measured on agar plates and in shaking cultures. The growth of bacteria could be quantified using a flatbed scanner or a cell phone camera. The majority of measurement and fabrication procedures have been replicated by low-skilled personnel (high-school students) in a low-resource environment (high-school classroom). Accordingly, this platform can be used as both an educational tool and as a diagnostic tool in low-resource environments worldwide.

Dr. Marc Dusseiller
Dusjags labs, hackteria.org, Switzerland

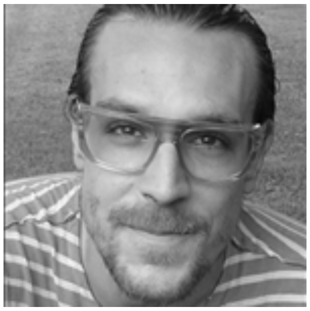

Dr. Marc R. Dusseiller is a transdisciplinary scholar, lecturer for micro- and nanotechnology (FHNW, University of Applied Sciences and Arts Northwestern Switzerland), cultural facilitator and artist. He works in an integral way to combine science, art and education. He performs DIY (do-it-yourself) workshops in lo-fi electronics, hardware hacking, microscopy, music and robotics. He is co-organizing Dock18, Room for Mediacultures, DYI* festival (Zürich, Switzerland), KIBLIX 2011 (Maribor, Slovenia), workshops for artists, schools and children as the president of the Swiss Mechatronic Art Society, SGMK
Currently, he is developing means to perform bio- and nanotechnology research and dissemination (Hackteria | Open Source Biological Art) in a DIY fashion in kitchens, ateliers and in developing countries.

Abstract:

BIOHACKING - DEMOCRATIZATION THROUGH DEMYSTIFICATION OF BIO- AND NANOTECHNOLOGY

Marc Dusseiller

hackteria.org | Open Source Biological Art, Zürich, Switzerland
Email: marc@dusseiller.ch

We will conclude from experiences in the framework of "Hackteria | Open Source Biological Art" and in educational projects with science students, game-designers, artists, hackers and children. During these transdisciplinary collaborations and workshops we have developed a rich online resource of instructions to build DIY (do-it-yourself) laboratory infrastructure using hacked consumer electronics and also novel methods of cultivating creativity across disciplines and public dissemination of Bio- and Nanotechnology.

http://hackteria.org/

Dr. Catherine Klapperich
Boston University, Boston, MA, USA

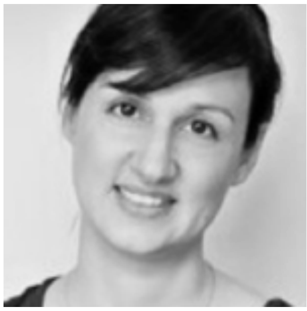

Dr. Catherine Klapperich is a Kern Innovation Faculty Fellow and an Associate Professor of Biomedical Engineering at Boston University. She is the Director of the Biomedical Microdevices Laboratory and is also a member of the Center for Nanoscience and Nanotechnology. Before coming to Boston, Dr. Klapperich was a Postdoctoral Fellow at Lawrence Berkeley Laboratory in the lab of Dr. Carolyn Bertozzi, and was a Senior Research Scientist at Aclara Biosciences in Mountain View, CA. She earned her Ph.D. in Mechanical Engineering in 2000 from the University of California, Berkeley with Drs. Lisa Pruitt and Kyriakos Komvopoulos; her M.S. in Engineering Sciences from Harvard University and her B.S. in Materials Science and Engineering from Northwestern University.

Abstract:

LOW COST DIAGNOSTICS FOR MONITORING DRUG REGIMEN ADHERENCE

Catherine Klapperich

Department of Biomedical Engineering, Boston University, 44 Cummington Street, Boston, MA 02215, United States
Email: catherin@bu.edu

The Klapperich Laboratory for Appropriate Healthcare Technologies at Boston University is focused on the design and engineering of minimally instrumented, disposable systems that enable low-cost point-of-care molecular diagnostics. We have invented technologies to perform microfluidic sample preparation for bacterial and viral targets from several human body fluids including, urine, blood, stool and nasowash. We are currently working on devices for the detection of infectious diarrhea, influenza, HIV, gonorrhea, chlamydia, MRSA and cancer biomarkers. At this conference, we will present a minimally instrumented, lab free system for preparing, storing and shipping nucleic acids from field sites to central labs.

Dr. Doug Weibel
University of Wisconsin, Madison, WI; USA

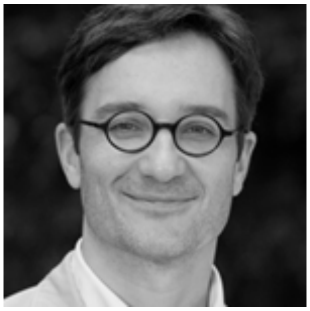

Douglas B. Weibel received his B.S. degree in chemistry in 1996 from the University of Utah (with Prof. C. Dale Poulter). From 1996-1997 he was a Fulbright Fellow at Tohoku University, Japan where he studied the organometallic chemistry of Pd complexes (with Prof. Yoshinori Yamamoto). He received his M.S. (1999) and Ph.D. (2002) from Cornell University (with Prof. Jerrold Meinwald) for research in the fields of organic and analytical chemistry. From 2002-2006 he was a postdoctoral fellow with Prof. George M. Whitesides at Harvard University. He is currently an Assistant Professor of Biochemistry (and Biomedical Engineering), an affiliate of the Genome Center, and a trainer in the Biophysics Program, Biotechnology Training Program, the Cellular and Molecular Biology Program, the Chemistry and Biology Program, the Materials Science Program, the Molecular Biosciences Training Program, and the Microbiology Doctoral Training Program, all at the University of Wisconsin-Madison.

Abstract:

BACCHIP: PORTABLE HANDS-FREE SYSTEM FOR BACTERIAL IDENTIFICATION AND TREATMENT

Doug Weibel

Department of Biochemistry, University of Wisconsin-Madison, 433 Babcock Drive, Madison, WI 53706, United States
Email: weibel@biochem.wisc.edu

We are developing portable diagnostic devices for detecting and treating microbial infections in resource-poor settings. These systems do not contain moving parts nor do they require power for their operation. They consist of a degassed layer of polymer that is preloaded with reagents and antimicrobial agents and enables the simultaneous identification of microbial pathogens and their susceptibility to clinical antimicrobial agents. The devices are inexpensive, are small enough to fit in a pocket, and have a shelf life of several months. Importantly, they require only a single step to load and operate, which minimizes the requirement for technical training. The time-to-detection rivals large, expensive clinical instruments that represent the gold-standard in this field.

Dr. Jonas Tegenfeldt
Lund University, Sweden

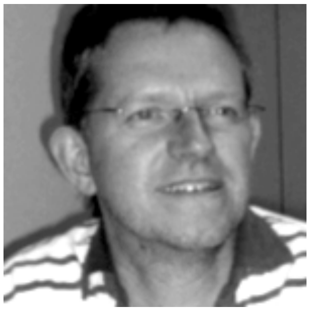

Dr Tegenfeldt is an associate professor in Physics at Lund University and University of Gothenburg in Sweden. His main research focus is on developing micro and nanofluidics tools for fundamental studies and applications with relevance for biomedicine. Having a basic training in Engineering Physics (MSc, Uppsala University) he continued to persue his PhD in Nanotechnology (PhD, Lund University). Later on he spent his postdoc in the labs of Bob Austin and Ted Cox at Princeton University before returning to Sweden to establish his own group. During his career Dr Tegenfeldt has pioneered the use of electrodeless dielectrophoresis, direct visualization of DNA stretched in nanofluidic channels and recently morphological sorting using deterministic lateral displacement. Currently, his group focuses on a wide range of topics including fundamental polymer physics, advanced labeling schemes for DNA, and deformability based sorting and morphology based sorting of cells with special emphasis on pathogen enrichment for diagnosis.

Abstract:

PATHOGEN ENRICHMENT USING DEFORMABILITY AND MORPHOLOGY BASED SORTING

Jonas Tegenfeldt

Division of Solid State Physics, NmC at LU, Lund University, PO Box 118, S-221 00 Lund, Sweden
Email: jonas.tegenfeldt@ftf.lth.se

Diagnosis of many parasitic disease such as sleeping sickness largely relies on direct observation of the pathogen. For sleeping sickness the relevant parasitemia is however so low that an enrichment is absolutely necessary to be able to find the parasites within reasonable time in a microscope. Using microfluidic sorting devices based on deterministic lateral displacement and made sensitive to the difference in morphology between the erythrocytes and the parasites we have shown proof of principle of extraction of parasites from whole blood. Our technique can be extended so that it is sensitive to the deformability of the cells as well, which in turn may be relevant for extraction of malaria infected erythrocytes.

Dr. Hywel Morgan
University of Southampton, UK

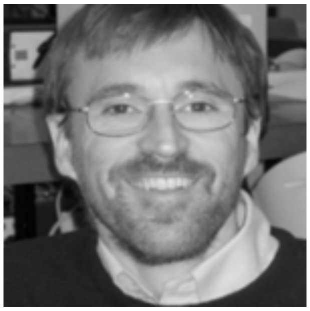

Hywel Morgan has a degree in Electronic Engineering and a PhD in biophysics from the University of Wales in 1985. In 1993 he moved to the University of Glasgow as a lecturer and in 2003 moved to the University of Southampton as Professor of Bioelectronics. He is a Fellow of the Institute of Physics and a Fellow of the Royal Society of Chemistry. His research interests are in electrokinetics and in developing micro-analytical systems for diagnostics and environmental monitoring.

Abstract:

THE POINT OF CARE COMPLETE BLOOD COUNT

Hywel Morgan

School of Electronics and Computer Science, University of Southampton, Southampton, SO17 1BJ, United Kingdom
Email: hm@ecs.soton.ac.uk

We are developing a microfluidic technology for point of care and patient self-testing of a complete blood count. The miniature cytometer is based on a compact sample processing cartridge with cell analysis chip and reader format. The technology is capable of performing a 3 part differential white blood cell count, platelet count, red blood cell count and haemoglobin measurement using a small sample (uL) High sensitivity and low co-incidence can be achieved without the need for sheath flow or optical scattering techniques that are used in main stream haematology analyzers. Cell counts in whole blood are performed in two parallel channels, one for red blood cells and platelets and another for a three-part differential white blood cell count with an assay time of a few minutes. Concordance tests on clinical samples show excellent correlation against large scale commercial blood analysis equipment. The technology can be further extend for other cell characterization assays such as CD4+ counting for HIV.

Dr. Michelle Khine
University of California, Irvine, CA, USA

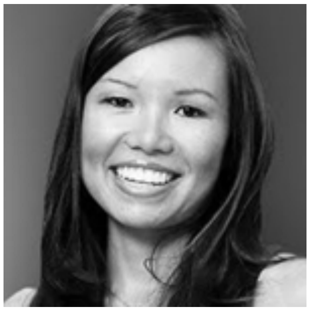

Michelle Khine is currently an Associate Professor of Biomedical Engineering, Mechanical Engineering, Chemical Engineering and Materials Science at UC Irvine. She was an Assistant & Founding Professor at UC Merced ('06-'09). Michelle received her BS and MS from UC Berkeley in Mechanical Engineering ('99 and '01, respectively) and her PhD under Luke P Lee in Bioengineering ('05) from UC Berkeley and UCSF. She is the Scientific Founder of Fluxion Biosciences and Shrink Nanotechnologies. Michelle was the recipient of the TR35 Award and named one of Forbes '10 Revolutionaries' in 2009 and by Fast Company Magazine as one of the '100 Most Creative People in Business' in 2011. Most recently, she was awarded the NIH New Innovator's Award, was named a finalist in the World Technology Awards for Materials, and was named by Marie-Claire magazine as 'Women on Top: Top Scientist'.

Abstract:

SHRINK-INDUCED MANUFACTURING PROCESS FOR LOW COST DIAGNOSTICS (SIMPL-CD)

<u>Michelle Khine</u>

Department of Biomedical Engineering, University of California, Irvine, CA
Email: mkhine@uci.edu

The challenge of micro- and nano-fabrication lies in the difficulties and costs associated with patterning at such high resolution. Instead we pattern at the large scale, which is easy and inexpensive, and rely on the heat-induced relaxation of pre-stressed polymer sheets – commodity shrink-wrap film – to achieve our desired structures. Using this approach, we have demonstrated that we can create fully functional and complete microfluidic devices with integrated nanostructures, printed electronics, and even optical components, all within minutes. These devices can be created for only pennies per chip and without any dedicated costly equipment. Because this process is compatible with roll-to-roll plastic processing, it is also scalable and cost-effective enough for point of care applications.

Dr. William Shu
Heriot-Watt University, Edinburgh, UK

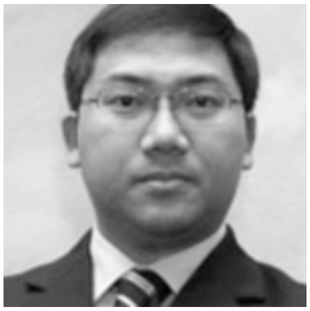

Dr Will 'Wenmiao' Shu is a Lecturer in Microengineering at Heriot-Watt University, Edinburgh. Originally from China, he received his Ph.D. in electrical engineering from Cambridge University Engineering Department and Nanoscience Centre. Then he worked as a postdoctoral research associate at Cambridge developing micro- and nano- sensor devices. In 2007, he joined as a lecturer in microengineering at the School of Engineering and Physical Sciences of Heriot-Watt University. He is also jointly appointed by the Edinburgh Research Partnership (ERP) Joint Research Institute of Integrated Systems (JRI -IIS), in collaboration with Edinburgh University. More recently, he has been a visiting scholar at Stanford University. His research interests focus on MEMS-based Biosensors, Lab-on-a-chip Systems and Biofabrication. His research group-Biomedical Microengineering Group develops innovative micro and nanotechnologies for point of care diagnostics, quantitative biology, synthetic biology and personalized medicine.

Abstract:

MANUALLY ACTUATED MICROFLUIDIC DEVICE FOR BLOOD PLASMA SEPARATION

William Shu

School of Engineering & Physical Sciences; Mechanical Engineering, NS 2.09, Heriot-Watt University, Edinburgh, EH14 4AS, United Kingdom
Email: w.shu@hw.ac.uk

We have developed a simple and low-cost microfluidic device for blood plasma separation. In order to detect disease biomarkers from whole blood sample, high-efficiency blood plasma separation is required. Although several types of microfluidic devices have been demonstrated, most approaches rely on high precision microchannels, addition of chemicals and a syringe or vacuum source. We developed a novel microfluidic device that is able to efficiently separate plasma from blood using a simple one-step manual process. The device is low-cost and desirable for low-resource, point-of-care diagnostics.

Dr. Jonathan Cooper
University of Glasgow, UK

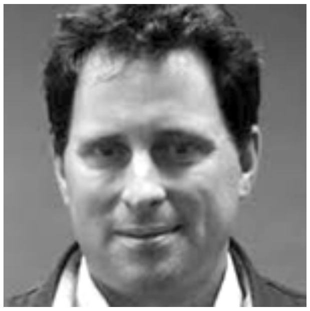

Dr. Jonathan Cooper is Head of the Division of Biomedical Engineering, in the School of Engineering and sits on the School's Management Board. He developed the first undergraduate degree in Biomedical Engineering in Scotland, with major teaching contributions from Engineering, Biology and Medicine, as well as Glasgow's hospitals. Jon is also the Dean of Graduate Studies in the College of Science and Engineering and sits on the College Management Board. He also leads Glasgow University's Doctoral Training Centre in Cell and Proteomic Technologies, funded by EPSRC and BBSRC.

He is also the founder of Mode Diagnostics (www.modedx.com), producing home diagnostics for bowel cancer and other bathroom diagnostic tests (and is currently involved in shaping an exciting joint venture between Mode-Dx and the China Bowel Cancer Screening Service). He is also involved in founding two other University Spin-Outs (in progress), in Developing World Diagnostics and in Drug Discovery Tools.

He has helped build University collaboration with Tianjin and Nankai Universities (China); NTU, NCKU and NTHU (Taiwan); A*Star and Nanyang Technical University (Singapore); as well as Mahidol University (Thailand). This broad internatonal activities have provided numerous opportunities for the exchange of Post Graduate Students.

Jon was elected as a Fellow of the Royal Academy of Engineering (2004) and a Fellow of the Royal Society of Edinburgh (2001). He was appointed to the Wolfson Chair in Biomedical Engineering in 2009 and was awarded a Royal Society Merit Award in 2010. He served on RAE'08 (EEE) and will serve on REF'14 (General Engineering).

Abstract:

TBA

Jonathan Cooper

University of Glasgow, Dept. of Electrical Engineering, Glasgow G12 8QQ, Scotland
Email: Jon.Cooper@glasgow.ac.uk

The "neglected diseases" such as sleeping sickness have a devastating impact on rural communities, in sub-Sahara Africa. The levels of infection within individuals are sufficiently low that point of care instruments must show extra-ordinary sensitivity. We show that low power cell separation methods, based upon electrical and-or acoustic fields, can be combined with mobile phone technologies to enrich and detect micro-organisms at appropriate levels (50 parasites/ml)

Dr. Charles Mace
Diagnostics For All (DFA), Cambridge, MA; USA

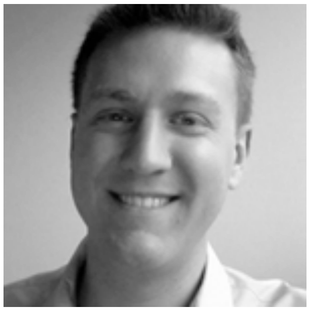

Charles Mace is a Senior Scientist at DFA. He received a B.S. in Physics from Le Moyne College in 2003 and his Ph.D. in Biophysics from the University of Rochester in 2008, under the supervision of Benjamin L. Miller. From 2008 to 2011, he was a postdoctoral researcher at Harvard University under the supervision of George M. Whitesides. His research interests include the development and implementation of simple healthcare technologies that provide clinically actionable data at the point-of-care.

LOW-COST DIAGNOSTICS TO IMPROVE SMALL FARMER INCOME IN RESOURCE-CONSTRAINED SETTINGS

Charles Mace

Diagnostics For All, 840 Memorial Drive, Cambridge, MA 02139, USA
Email: cmace@dfa.org
Phone: +1-617-494-0700

Resource-constrained settings have limited access to the tools, methods, and expertise required to maximize the earning potential of small-scale, sole-proprietor farms. As a result, proper testing procedures for agricultural goods (e.g., the safety of consumables or the health livestock) are frequently disregarded or falsified. We are developing a suite of low cost, rapid, point-of-care diagnostic assays that are aimed at improving economic outcomes for small shareholder farmers by providing them with tools to better manage their dairy herds and improve the quality of their dairy and maize products. The use of these assays will protect human and animal health and, by assuring the quality of products, increase the incomes of farmers.

Ashok A. Kumar (A.J.)

Whitesides Lab, Harvard University, Cambridge, MA

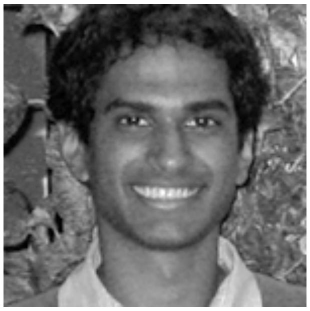

Ashok (A.J.) Kumar, is a PhD candidate in Applied Physics working with George Whitesides and Robert Westervelt at Harvard University. After completing his B.S. in Physics at Stanford University, he served as a U.S. Peace Corps Volunteer in rural South Africa. Combining his interests in social development and science, his work focuses on creating novel techniques to analyze blood in low-resource settings

Abstract:

DIAGNOSTICS BY DENSITY

Ashok A. Kumar

12 Oxford St., Mallinkrodt Bldg. Rm. 251, Cambridge, MA 02138, USA
Harvard University
Email: akumar@gmwgroup.harvard.edu

Aqueous multiphase systems (MuPSs) of polymers and surfactants provide self-assembling step-gradients in density. The interfaces between liquid phases are thermodynamically stable and define a step in density, which can be as small as $\Delta\rho = 0.001$ g/cm^3. When a cell sediments through a MuPS under centrifugation, it will collect at an interface between phases that brackets its density. This technique allows blood cells to be rapidly sorted by density and provides important hematological data. The proper choice of densities allows enrichment of cells affected by diseases such as malaria and sickle cell disease.

Density provides a physical marker for cells. Using the unique, self-establishing density steps formed by mixtures of immiscible solutions of aqueous polymers, we are developing tests to do basic hematology tests as well as to detect diseases and disorders that affect cell densities, such as malaria and sickle cell disease.

Dr. Reginald Beer
National Security Engineering Division, Center for Micro- and Nanotechnologies, Lawrence Livermore National Laboratory

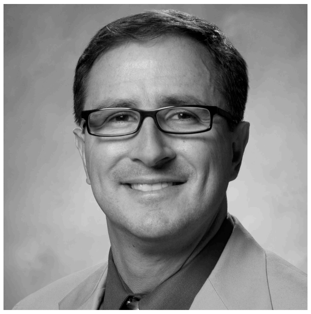

Dr. Reginald Beer is a Medical Diagnostics Initiative Leader who develops Lab-on-Chip technologies for time-critical Molecular Diagnostics applications. His interest in real-time detection is focused on supplying the physician or emergency responder with tools for rapid and accurate diagnostics under harsh conditions. His research and publications in digital PCR, on-chip miniaturization, and rapid amplification have lead to several new technologies for improved diagnostics applications and outcomes.

Abstract:

COOPERATIVE THREAT REDUCTION FOR THE 21ST CENTURY

Reginald Beer

Center for Micro- and Nanotechnologies, Lawrence Livermore National Laboratory, 7000 East Avenue, Livermore, CA 94550
Email: beer2@llnl.gov

The global reach of the U.S. military increases the likelihood of civilian or military personnel to the exposure to emerging infectious diseases and pandemics. Since the 1970s, new emerging infectious diseases have been identified at the unprecedented rate of one or more per year. Many naturally occurring infectious diseases and pathogens are well-suited for weaponization by an adversary for biological warfare. Effective public health systems to counter are essential to mitigating and preventing risks from biological threats. The global health security and biodefense nexus represents an opportunity to define shared objectives for threat reduction activities because the same tools required to prevent, detect, assess, report, and respond to infectious disease outbreaks will increase the ability to counter biological threats whether natural, accidental or deliberate. An unexplained but contained outbreak of anthrax might be traceable to natural causes or deliberate release, either of which would require the same suite of medical countermeasures and defense capabilities. More importantly, the global health security and biodefense nexus offers a clear and compelling rationale for sustained-engagement and cooperation with international partners. Promoting global health security is a shared responsibility that requires global cooperation, sustained-engagement, and enabled by low-cost, deployable technologies.

Ms. Nana Yaa Boadu

Univerisy of Alberta, Edmonton, AB; Canada

Abstract:

RAPID MALARIA DIAGNOSIS IN GHANA: IMPLEMENTING POLICIES, AND NAVIGATING TECHNOLOGY AT THE POINT OF CARE

Boadu N.Y.*[1], Ansong D.[2], Higginbottom G.[3], Einsiedel E.F.[4], Yanow S.K.[1,5]

[1]*School of Public Health, 3-300 Edmonton Clinic Health Academy, 11405 87 Avenue, University of Alberta, T6G 1C9, Canada. Email: boadu@ualberta.ca*
[2]*Department of Child Health, School of Medical Sciences, Kwame Nkrumah University of Science and Technology, Kumasi, Ghana. Email: ansongd@gmail.com*
[3]*Faculty of Nursing, University of Alberta, 4-171 Edmonton Clinic Health Academy, 11405 87 Avenue, University of Alberta, T6G 1C9, Canada. Email: gina.higginbottom@ualberta.ca*
[4]*Department of Communication and Culture, University of Calgary, Alberta, T2N 1N4, Canada. Email: einsede@ucalgary.ca*
[5]*Research and Development – ProvLab, WMC 2B4.59, 8440 112 Street, Edmonton, AB, T6G 2J2*
**Corresponding Author*

Background: Recently adapted World Health Organization (WHO) policies in Ghana recommend testing before treating all suspected malaria patients above five years of age. The original WHO guidelines refer to patients of all ages. Rapid diagnostic tests (RDTs) allow for quick and reliable diagnosis of malaria in peripheral facilities with limited laboratory capacity, without having to rely exclusively on clinical diagnosis. Implementing national health policies in limited-resource settings can be challenging for frontline health services workers. Such challenges may be amplified where implementation involves the uptake of new technology, such as RDTs. Studies reveal variable extents to which test results are followed for guiding fever case management across sub-Saharan Africa. Little is known about health workers' strategies for integrating RDT-use with fever case management in resource-constrained environments.
Objectives: 1) to examine national guideline adherence among health workers performing rapid malaria diagnosis; 2) to investigate health workers' strategies for point-of-care malaria testing with RDTs; and 3) to understand how health workers integrate policy with practice amidst limited resources.
Methodology/methods: A focused ethnography involving rural/peri-urban district health workers in direct observations, interviews and focus group discussions on rapid diagnostic testing for malaria between April and June 2012.
Results: This paper presents early findings on how health workers in this study integrate RDT-use and applied policies for rapid malaria testing in their facilities. Lessons drawn will be useful for informing optimization strategies in the application of RDTs and similar point-of-care diagnostic technologies in developing country health settings.

Mr. Daniel Maina

Aga Khan University Hospital, Nairobi, Kenya

Abstract:

CHALLENGES IN ESTABLISHING POINT OF CARE TESTING IN A DEVELOPING COUNTRY; THE AGA KHAN UNIVERSITY HOSPITAL EXPERIENCE

Daniel Maina
Aga Khan University Hospital, Nairobi

The AKU hospital committee is tasked with the responsibility of overseeing the formulation and implementation of Point of Care Testing policy within the hospital. Amongst the challenges faced by the committee are issues such as training the end-users on quality assurance, method validation, and the fact that we don't have national guidelines or a regulatory framework to regulate POCT and/or equipment used across various institutions.

Dr. Martin Mwangi Thuo

Harvard University, Cambridge, MA

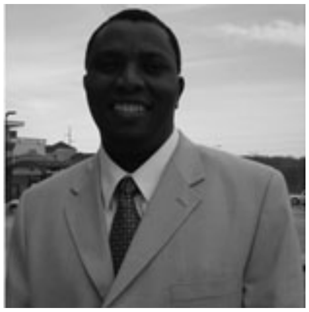

Dr. Martin Thuo received a doctorate in organic chemistry from University of Iowa in 2008. Dr. Thuo is an alum of Kenyatta University. From Feb. 2009, he has been a postdoctoral fellow at Harvard University under the mentorship of prof. George Whitesides. Since 2010 he has also been an adjunct faculty in chemistry at Kenyatta University. His research interests are in applied soft materials with a special focus on low cost devices and surface/interface studies.

Abstract:

LOW-COST MICROFLUIDIC DEVICES DERIVED FROM ULTRA-HYDROPHOBIC PAPER

<u>Martin Mwangi Thuo</u>

Harvard University, Cambridge, MA
Email: mmwangi@gmwgroup.harvard.edu

We describe the development of open channel microfluidic devices and well-plates constructed using ultra-hydrophobic paper as the structural material. The working principle on which these paper-based devices are based is the almost superhydrophobicity generated by covalent grafting of non-polar hydrocarbons on a paper substrate. The elasticity of the paper allows us to emboss different channel patterns on a paper substrate. We can also generate analogous patterns by cutting and stacking the layers of paper and tape. These devices are low-cost and fabrication can be done in a few hours. We discuss the fabrication and application of these paper-based microfluidic devices (see figure below) in crystal growth and fluid flow. We also observe laminar flow with two or three fluids and demonstrate that these devices are analogous to regular PDMS-based devices. These devices can find uses in bio-analytical, diagnostics, genomics, and chemistry laboratories among others.

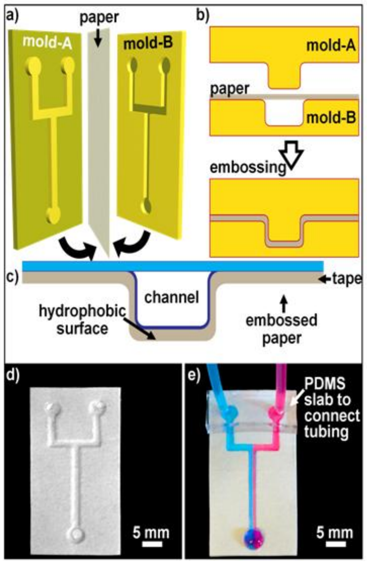

Ms. Claire Mugasa

Makerere University, Kampala, Uganda

Abstract:

COMPARISON OF NUCLEIC ACID SEQUENCE-BASED AMPLIFICATION AND LAMP FOR DIAGNOSIS OF SLEEPING SICKNESS

Claire M. Mugasa[1], Enock Matovu[1], Diana Katiti[1], Alex Boobo[1], George W. Lubega[1], Henk D.F.H. Schallig[2,]

[1]School of Biosecurity, Biotechnical and Laboratory Sciences, COVAB, Makerere University Kampala, Uganda., [2]Koninklijk Instituut voor de Tropen (KIT)/Royal Tropical Institute, KIT Biomedical Research, Amsterdam, The Netherlands.

Molecular diagnostic tests are paramount especially in diagnosis of *Trypanosoma brucei gambiense* infections where the parasitaemia is usually very low. In this study we compare two simplified molecular tests for the diagnosis; for nucleic acid sequence-based amplification (NASBA) and loop- mediated isothermal amplification (LAMP) of clinical samples from patients and serological suspects of *T. b. gambiense* infection in Uganda. A total of 116 samples were analyzed and the diagnostic sensitivity and specificity of NASBA was 90.6% (95%CI; 80.7-96%) and 100% (95%CI; 94.9-100%), while the sensitivity and specificity for LAMP as compared to microscopy as the reference test was 79.7% (95%CI; 68.2-87.9%) and 100% (95%CI; 91.6-100%) respectively. Both index test showed a highly significant statistical difference in accuracy when compared to microscopy (P=0.0001). Of the suspect samples tested, 4 (13.3%) and 18 (60%) of the samples tested positive with NASBA and LAMP respectively, and 3 of these samples gave a positive result in both NASBA and LAMP. LAMP and NASBA-OC showed comparable diagnostic accuracy when used to diagnose HAT. Larger studies are recommended to explicate the function of these tests in detecting CATT suspect samples and follow-up of such to parasitological conversion of the suspects.

Mr. Christopher Kariuki
Institute of Primate Research, Nairobi, Kenya

Abstract:

DEVELOPMENT OF A NANOBODY® BASED DIAGNOSTIC TOOL FOR *TRYPANOSOMA BRUCEI RHODESIENSE*

Christopher Kariuki, John Kagira, Simon Karanja, Naomi Maina and Maina Ngotho

Institute of Primate Research, Karen, Nairobi, Kenya; JKUAT, Kenya

Human African trypanosomiasis (HAT) is a vector borne parasitic disease. It is considered a neglected re-emerging disease of public health importance affecting mainly the poor in remote rural African communities. The diagnosis, particularly the stage determination of the disease is critical in the choice of therapeutic intervention. There is need for improvement of diagnostic methods as parasite detection remains insufficiently sensitive and cumbersome. Up to now, a simple serological field applicable diagnostic test for T. b. rhodesiense infection is yet to be implemented. Nanobody® (miniature antibody) technologies are a recent discovery in the field of disease diagnosis. Derived from an *in vivo* matured camelid immune cell complementary DNA (cDNA), nanobodies have high potential for diagnostic applications in HAT. These recombinant miniature antibodies can be engineered against the conserved regions of variable surface coat antigens on the trypanosome and, conjugated to molecules to increase their sensitivity.

Ms. Lucy Ochola
Institute of Primate Research, Nairobi, Kenya

Abstract:

MALARIA DIAGNOSIS: NEED TO TRACK THE CHANGING FACE OF MALARIA TRANSMISSION

Lucy Ochola[1], Thomas Kariuki[1], Bernhards Ogutu[2]

[1]*Institute of Primate Research, Nairobi, Kenya,* [2]*Centre for Clinical Research, Kenya Medical Research Institute*

Increased investment in malaria research and control leading to improved distribution of insecticide-treated bednets and introduction of artemesinin derivatives in the last two decades has resulted in an overall reduction in the incidence of malaria disease. This has been coupled with a reduction in transmission levels making malaria elimination a possibility. However, complete control and eradication cannot be achieved within a framework of poor diagnostic techniques and policies that tend to ignore low grade parasitaemia especially in asymptomatic carriers that provide a pool for transmission of malaria. The current techniques for malaria diagnosis include microscopic examination of peripheral blood films, antigen detection using rapid diagnostic tests (RDTs) and molecular based methods. RDTs and microscopy may not be reliable especially when parasitaemia fall below 5-10parasites/µl, while molecular methods have a sensitivity and specificity of 100% at 1 parasite/µl but are yet to be developed for point of care use. Microscopy is subjective and heavily relies on the proficiency of the user. Furthermore, there is evidence that RDTs may not be very sensitive especially in varying transmission intensities and ecological settings. We discuss the importance of diagnostic techniques in the fight to eradicate and control malaria and foreseen challenges and possible solutions.

Ms. Imna Malele
Tsetse & Trypanosomiasis Research Institute, Tanga, Tanzania

Abstract:

IMPORTANCE OF RAPID AND SENSITIVE DIAGNOSTIC IN ACCURATE ESTIMATION OF HUMAN AFRICAN TRYPANOSOMIASIS MAGNITUDE IN ENDEMIC AREAS IN TANZANIA

Malele, I.I.[1], H. Nyingilili[1], E. Lyaruu[1] & W. Kitwika[2]

[1]*Tsetse & Trypanosomiasis Research Institute (TTRI), Box 1026 Tanga, TANZANIA*
[2]*Tsetse & Typanosomiasis Research and Control Centre, Box 1380, Kigoma, Tanzania*

Sleeping sickness is one of the neglected tropical diseases. More than 60% of the total cases of Human African trypanosomiasis (HAT) reported locally originate from western Tanzania. Cases from Serengeti Ecosystem are reported mostly among tourists. HAT cases, are directly related to the prevalence of human infective trypanosomes in the vectors or host animals. Traditional detection microscopically have short comings as the method doesn't discriminate between human and animal disease causative parasites and is less sensitive. Reliance on such data, tend to underestimate the actual prevalence and thus the risks of HAT. Molecular diagnostic tools were incorporated in the analysis of vector samples collected from 2009 to 2011. Trapped tsetse were dissected and infected and uninfected organs were further analysed by SRA LAMP. The infection rate in tsetse salivary glands was 1/36 microscopically and 2/36 by SRA LAMP in 2009. In 2010 and 2011, microscopic infection was 9/36 and all were positive by SRA LAMP; and 11/50 positive microscopically but 7/50 positive by SRA LAMP respectively. The result by SRA LAMP confirms the presence of human infective trypanosomes circulating in the area and calls for the integration of rapid and sensitive diagnostic techniques in accurate estimation of the magnitude of HAT causative parasites and the risks posed by HAT. Rapid and diagnostic techniques are needed to revolutionize diagnostic of HAT in poor resource settings in rural areas.

Ms. Elizabeth Ochola
Kenya medical research institute, Kisumu, Kenya

Abstract:

A COMPARISON OF DIAGNOSTIC METHODS AGAINST PCR FOR THE DETECTION OF *S. MANSONI* AMONG SCHOOL CHILDREN IN WESTERN KENYA

Elizabeth A. Ochola[1], Daniel O. Matete[1], Karen T. Foo[2], Pauline N. M. Mwinzi[1], John M. Williamson[1], Susan P. Montgomery[2], Eric Brienen[3] , Jaco J. Verweij[3], L. VanLieshout[3], W. Evan Secor[2], and Diana M. S. Karanja[1].

1 Center for Global Health Research, Kenya Medical Research Institute, P.O. Box 1578-40100, Kisumu, Kenya;
2 Division of Parasitic Diseases and Malaria, Centers for Disease control and Prevention,1600 Clifton Rd, N. E.,Mailstop D-65, Atlanta,
3 Leiden University Medical Center, Department of Parasitology (ZoneP4-P) Albinusdreef 2 2333 ZA Leiden, The Netherlands

The most widely used tools for diagnosis of S. mansoni, stool examination and serologic detection, are limited by low sensitivity or inability to distinguish between current from former infections, respectively. As a result, there is not an accepted “gold standard” for S. mansoni diagnosis for evaluation of new diagnostic tools. However, recent development of a semi-quantitative PCR that detects schistosome DNA stool provides a tool with increased sensitivity and specificity for this purpose. We utilized the PCR method to help evaluate two versions of a point of contact (POC) test designed to detect circulating cathodic antigen (CCA) in urine of people infected with schistosomes. 1898 school children aged between 8-12 years from Rarieda District of western Kenya took part in the study. Three stool and three urine samples were collected on consecutive days for testing by Kato-Katz and the CCA cassettes. A portion of the first day’s stool was preserved in ethanol and subsequently tested for presence of schistosome DNA by PCR.In addition, serum from a single blood sample was tested by ELISA for anti-schistosome IgG. Children who were infected with S. mansoni were treated using praziquantel. Results: Compared to PCR, the initial version of the POC urine test (CCA-M) on any of the 3 days had the highest sensitivity at 92.11%, followed by Kato=-Katz at 70.44%. The alternate version of the POC test (CCA-A) and ELISA had sensitivities of 52.56% and 50.16%, respectively. This results show that PCR is a promising gold standard assay that can be used when evaluating the performance of different tests used for the diagnosis of S.mansoni infection present in Schistosomiasis endemic areas.

List of Posters

Poster	Presenter	Title
1	Abigal Weaver	Paper Analytical Devices (PADs) for Inexpensive Pharmaceutical Testing in Developing Countries
2	Allan Ole Kwallah	Novel assays for Yellow Fever Virus a zoonotic disease
3	Anne Fischer	ArboVirus Incidence and Diversity
4	Anne Nalunkuma Kazibwe	Red blood cell lysis method for parasite concentration in Human African Trypanosomiasis (HAT)
5	Areba Gerald	Antimalarial activity of small molecular weight inhibitors of Heat Shock Proteins (Hsps)
	Austin Aluoch	
6	Caroline Wasonga	Development of replenishable ELISA reagents for the detection of Chikungunya viral infection
7	Chris Kinyanjui	Development of a nanobody® based diagnostic tool for *Trypanosoma brucei rhodesiense*
8	Claire Mugasa	Comparison of nucleic acid sequence-based amplification and LAMP for diagnosis of sleeping sickness (**OP)**
9	Clémence Sicard	Paper-based colorimetric test strips
10	Daniel Maina	Challenges in establishing Point of Care Testing in a developing country; The Aga Khan University Hospital experience **(OP)**
11	Dawn Maranga	IL-6 as a marker for *Trypanosoma brucei rhodesiense* late stage disease in experimentally infected vervet monkey
12	Dennis Omondi	Diagnostics practicing veterinarians
13	Elizabeth Ochola	A Comparison of Diagnostic Methods against PCR for the detection of *S. mansoni* among school Children in Western Kenya.
14	Erick Atema	Effects of unregulated use of Glucometer on Management of Patients
15	Eugene Lyaruu	A study on the incidence of tsetse fly in relation to trypanosomosis prevalence and its impact on livestock production in Mpanda district – Tanzania
16	Faith Onditi	Baboon (Papio anubis) as a model for developing effective diagnostic tools for placental malaria in humans
17	Fredrick Maloba	Diagnostics for zoonoses
18	George Farquar	Diagnostics Evaluation and Performance Laboratory for Operational Yardsticks
19	Gizachew Bayleyegn	Tuberculosis in camels and humans in pastoral regions of Ethiopia (OP)
20	Hawa Mavura	Haematological changes in sheep (Ovis aries) infected with human infective Trypanosoma brucei rhodesiense
21	Imna Malele	**I**mportance of rapid and sensitive diagnostic in accurate estimation of Human African Trypanosomiasis magnitude in endemic areas in Tanzania (OP)

22	J.M. Nguta	Safety evaluation of traditionally used antimalarial plants
23	Jason Beech	Simple Analytical Devices Sensitive to the Size, Shape and Deformability of Cells
24	Jemimah Oduma	Effects of herbal medicines used by herbal practitioners on the reproductive process
25	Joerg Jores	Towards the development of an improved diagnostic test for Contagious Bovine Pleuropneumonia
26	Johnson Nasimolo	Potential utilization of natural products in the treatment of sleeping sickness
27	Julianne Muthinja	Genotyping of *Trypanosoma congolense* in cattle and Glossina pallidipes (tsetse flies) in shimba hills area, Kenya
28	Katrina Tjhung	Development of sensing platform for TB-specific antibodies using paper-based bacteria cultures and engineered bacteriophage
29	Kem Githui	Population genetics of *Plasmodium falciparum* and the host
30	Kevin Kamanyi	The impact on the longevity of *Anopheles gambiae* infected by drug resistant *Plasmodium falciparum*
31	Kirtika Patel	Diagnostics at Moi University School of Medicine
32	Lonzy Ojok	Establishment of diagnostic technologies for increased animal production in Uganda
33	Lucy Ochola	Malaria diagnosis: need to track the changing face of malaria transmission
9	Madiha Khan	Paper-based colorimetric test strips
34	Maina Ichagichu	Epidemiology of toxoplasmosis and identification of parasite genotypes infecting humans in Thika district, Kenya
35	Mechtilda Byamungu	Validation of molecular diagnostic techniques for african animal trypanosomiasis (aat) in tanzania
36	Meshack Obonyo	Diagnosis for Amoebiasis
37	Michael Ndung'u	Development of an Olive baboon model for Alzheimer's disease
38	Nana Yaa Boadu	Rapid malaria diagnosis in Ghana: implementing policies, and navigating technology at the point of care
39	Naomi Maina	Biomarkers for sleeping sickness in vervet monkeys experimentally infected with Trypanosoma brucei rhodesiense
40	Nicholas Kiulia	Diagnostics for rota virus
25	Nimmo Ngatiri	Towards the development of an improved diagnostic test for Contagious Bovine Pleuropneumonia
41	Oli Abate Fulas	Diagnostics at point of care in Ethiopia
42	Onkoba Nyamongo	Diagnostics for cerebral and placental malaria
43	Paul Ogongo	Analysis of *Schistosoma mansoni* candidate antigens as diagnostic targets for schistosomiasis **(OP)**
44	Paul Rajula	Western Province (Kenya) External Quality Assessment Scheme

45	Paul Scanlan	Manually actuated microfluidic device for blood plasma separation
46	Purity Nguhiu	Diagnosis of Cyclosporiasis in non-human primates
47	Roshini Samuel	Capillary platform for malaria detection from whole blood by hydrogel PCR
48	Ruth Nyakundi	Use of circulating anodic antigen (CAA) detection as a surrogate marker for determining worm and egg burden in schistosomiasis infections
49	Salame Ashur	Development of monoclonal antibodies for use in diagnosis of Rift Valley Fever Virus
50	Samuel Mwangi Njoroge	Molecular characterization of antimicrobial resistance on Enterobacteriaceae from severely malnourished children attending Mbagathi District Hospital
51	Shem Mutuiri	Molecular Characterization of the RANTES Gene Polymorphisms in Nairobi Province, Kenya; Potential for Personalized Medicine **(OP)**
52	Silas Kiruki	Diagnostic tool for detection of enteropathogenic bacteria and protozoa in water
53	Stefan Holm	A Simple, cheap and portable device for point-of-care diagnosis of Sleeping-sickness
54	Swaleh Sauda	Analytical chemistry for diagnosis and chemotherapy
55	Tendai Gadzikwa	TB Diagnostics at point of care in Zimbabwe
56	Thomas Musembi	Simple technologies for testing for fake drugs
57	Victor Alioni	The role of Regulatory T-cells in Pathogenesis of Trypanosomiasis in mouse model
58	Vincent Owino	Validation of Variable Surface Glycoprotein (VSG) for Diagnosis of African Trypanosomes
59	Virginia Gichuru	Population genetics of host and parasite genes involved in invasion of malaria
60	Wycliffe Wanzala	A comparative analysis of dual and no-choice repellency bioas l oil of *Tagetes minuta* against climbing response behavior of *phalus appendiculatus*

Poster Abstracts:

Poster 1:

PAPER ANALYTICAL DEVICES (PADS) FOR INEXPENSIVE PHARMACEUTICAL TESTING IN DEVELOPING COUNTRIES

Abigail Weaver

University of Notre Dame

The 15-50% prevalence of substandard and counterfeit medicines in developing countries1 unnecessarily increases morbidity and mortality for treatable diseases and favors the emergence of drug resistant disease organisms.2 We are developing field screening tools for pharmaceuticals based on Paper Analytical Devices (PADs). PADs cost less than $US 0.35 apiece to fabricate, as we will describe at the workshop. They can carry out analytically complex tasks, but they are user-friendly, transportable, do not need electrical power or batteries, and can be automatically read using only a cell phone. Our first PADs test for acetaminophen, artemisinin combination therapy (ACT) drugs, antibiotics and related excipients. Our group is working to expand the library of pharmaceutical analytes using both chemical tests and biochemical methods, such as the use of genetically engineered yeast. We are working to transition this technology from the lab to clinics and will begin field testing in Kenya in June of 2012.

1.Cockburn, R., Newton, P.N., Agyarko, E.K., Akunyili, D. & White, N.J. The global threat of counterfeit drugs: why industry and governments must communicate the dangers. PLoS medicine 2, e100 (2005).
2.Newton, P.N., Green, M.D., Fernández, F.M., Day, N.P.J. & White, N.J. Counterfeit anti- infective drugs. The Lancet infectious diseases 6, 602-13 (2006).

Poster 2:

NOVEL ASSAYS FOR YELLOW FEVER VIRUS A ZOONOTIC DISEASE

Allan Ole Kwallah

KEMRI-ITROMID

My PhD work involves the development of novel assays for Yellow Fever Virus a zoonotic disease. Hence accorded a chance to attend the workshop will enable me equip myself with the current treads in point of care diagnostics. In resource limited settings the provision of POC aids in averting outbreaks especially in viral heamorrhagic fevers once reliable tests are availed in disease prone zones.

Poster 3:

ARBOVIRUS INCIDENCE AND DIVERSITY

Anne Fischer

ICIPE

This work is the result of the AVID consortium, lead by Dr. Rosemary Sang. The consortium comprises:

-International Center for Insect Physiology and Ecology (*icipe*)
-International Livestock Research Institute (ILRI)
-Kenyan Medical Research Center (KEMRI)
-Department of Veterinary Services (DVS)
-Kenyan Wildlife Services (KWS)
-Kenyan Agricultural Research Center (KARI)
-Ministry of Public Health and Sanitation (MoPHS)

The worldwide emergence of arbovirus infections is unparalleled in East Africa where Yellow Fever, Dengue, RFV, Onyong-nyong, Crimean Congo Hemorrhagic Fever and Chikungunya viruses caused disease epidemics in humans in the recent past. The Arbovirus incidence and Diversity (AVID) project lead by icipe aims at strengthening regional capacity for disease surveillance and diagnostics. The project makes use of cutting edge high-throughput diagnostic and sequencing technologies to determine the circulation of arboviruses among human, livestock, wildlife and arthropod vectors in selected RVF epidemic-prone hotspots of Kenya. Samples are screened using family specific primers with MassTag PCR technology, a new technology PCR based on Mass Spectrometric detection of end product. Detection of specific virus is then done using High Resolution Melting. In addition, metagenomic analysis enabled us to assemble full viral genomes and to discover novel emerging pathogens in unexpected hosts. For example we were able to identify pigs as a potential reservoir for Ndumu virus.

Poster 4:

RED BLOOD CELL LYSIS METHOD FOR PARASITE CONCENTRATION IN HUMAN AFRICAN TRYPANOSOMIASIS (HAT)

Matovu Enock[1], Kazibwe Anne[1], Boobo Alex[1], Biéler Sylvan[2], Ndung'u Joseph[2]

[1] Department of Veterinary Parasitology and Microbiology, School of Veterinary Medicine, Makerere University, P.O. Box 7062,Kampala, Uganda
[2] Foundation for Innovative New Diagnostics (FIND), 71 avenue Louis-Casaï, P.O. Box 93, 1216 Cointrin, Switzerland

Available parasitological tests in diagnosis of HAT have limited sensitivity, frequently leading to misdiagnosis of patients with the commonly low parasitaemia. In such cases, minority trypanosomes are shielded by the host red blood cells (RBCs). Yet accurate diagnosis and staging that rely on these tests are crucial in the successful control of HAT. We explored the potential of selective lysis of RBCs for enhancing the parasite detection. Mouse blood spiked with known numbers of trypanosomes was incubated in a home-made ammonium chloride solution or commercially obtained RBC lysis solution. After 5 minutes, the mixtures were centrifuged and the pellets used to prepare wet smears for microscopy. These parasite concentration procedures increased the sensitivity up to 20 times compared to unlysed blood with the same numbers of trypanosomes, which remained motile for several hours in the lysis solutions. This method can be used to concentrate up to 5ml of blood. The reagents require no cold chain making it easy to incorporate this RBC lysis step into the HAT diagnostic algorithm. This could provide template for use with advanced parasite visualization tools, such as the LED fluorescence microscope or molecular diagnostics such as the loop-mediated isothermal amplification (LAMP) of DNA and PCR.

Poster 5:

ANTIMALARIAL ACTIVITY OF SMALL MOLECULAR WEIGHT INHIBITORS OF HEAT SHOCK PROTEINS (HSPS)

Areba Gerald[1], Rasheed Khalid[2], Victor Alioni[2]

1. Ministry of Livestock Development - Kenya and Department of Biochemistry and Molecular Biology, Egerton University, Njoro Campus- KENYA.
2. Department of Biochemistry and Molecular Biology, Egerton University, Njoro Campus, Nakuru-KENYA

Malaria presents one of the major health issues worldwide, with over 80% of all cases occurring in Africa. Several HSPs which are localized in the mitochondria are highly conserved and universally present molecular chaperones that protect cell structures and organelles against thermal, chemical and redox stress. These proteins play crucial roles in folding/unfolding/assembly of proteins, transport/sorting of proteins into correct subcellular compartments, cell cycle control, signaling, and antigen presentation. This is a protective system that is very essential for Plasmodium falciparum survival. Several antimalarials increase the oxidative stress and lead to up regulation of HSPs, therefore combination therapy with small molecular weight inhibitors of HSPs will enhance their effectiveness in killing the parasites or removal by phagocytosis, and In addition several inhibitors of HSPs are currently developed for cancer therapy and might be important as anti-malarials. There are Four major subclasses of HSPS viz; Hsp90, Hsp70, Hsp60, and small Hsps. This study will assess the antimalarial activity of small molecular weight inhibitors of heat shock proteins using three specific objectives: by determining the growth of *P falciparum* in presence of a combination of small molecular weight inhibitors of HSPs and the commonly used antimalarials (artemisinins, aminoquinolines and antifolates) in vitro, comparing the in vitro activity of antimalarial drugs and small molecular weight inhibitors alone and evaluating the potential of in vitro cross-reactions between the two drugs. Parasite isolates will be cultured using the candle jar technique, drug response assay carried out by subculturing the parasites in the presence of various antimalarial drug concentrations and small molecular weight inhibitors of HSPs, in vitro activity of small molecular weight inhibitors assessed as the drug concentration that inhibits 50% of parasite growth (IC50) using the SYBR green I microtest. Relative levels of HSPs mRNA levels will be measured using qRT-PCR while, relative levels of HSP proteins will be measured using western blots techniques. The non-parametric Kruskal Wallis and Dunn’s post test will be used to compare the in vitro activity of antimalarials with that of the small molecular weight inhibitors and Spearman correlation analysis to evaluate the potential of in vitro cross-reaction patterns of the two drugs. Bioinformatics’ (NCBI, PlamoDBand Swiss model) databases will be used to check for isolates, 3D structure, drugability, identity, similarity and homology of the HSPs with those of the host. The significance of the study will be the identification of new compounds that target HSPs and are additive to antimalarials that can be assayed invivo to determine their efficacy and for use in the treatment of malaria

Poster 6:

DEVELOPMENT OF REPLENISHABLE ELISA REAGENTS FOR THE DETECTION OF CHIKUNGUNYA VIRAL INFECTION

Carolyne O. Wasonga[1], Lillian A. Musila[1], Juliette R.Ongus[2], James H. Kimotho[3] , Rosemary C. Sang[1]

[1]*Center for Virus Research, Kenya Medical Research Institute P.O.Box 54628-00200 Nairobi, KENYA*
[2]*Medical Laboratory Sciences Department, Jomo Kenyatta University of Agriculture and Technology P.O. Box 6200 0200 Nairobi KENYA*
[3]*Production Unit, KEMRI P.O.Box 54840-00200, Nairobi KENYA*
Corresponding author: cwasonga@gmail.com/cwasonga@wrp-nbo.org

Chikungunya (CHIK) virus is a potentially deadly disease that periodically cause outbreaks and pose a huge threat due to their increased global re-emergence necessitating continuous surveillance of vectors, human and animals. CHIK fever is a viral disease transmitted to humans by the bite of infected Aedes aegypti mosquitoes. The most recent outbreaks have been reported from Indian Ocean islands and re-infection of the virus occurred in Re-union Island and India in March 2010. This study focused on the co-circulating variants of the Comorros CHIK virus. The virus titer and plaque morphology was determined using plaque assay and the E1 region of samples were amplified by RT-PCR, expected band size of 787bp was achieved by presence of bands on the 2% agarose gel. Phylogenetic analysis showed 3 strains from Comoros clustered together with other Comoros strains and were closely related to Re-Union Island and Lamu strains of CHIK. The other 2 strains clustered together with the CHIK S-27. The African strains were clustered separately from the Indian ocean islands, showing genetic variation as the outbreak spread from Lamu to Comoros then Re-uonion Island. Plaques of different sizes were observed suggesting a co-circulating strain/virus in the Comoros Island isolate.

Poster 7:

DEVELOPMENT OF A NANOBODY® BASED DIAGNOSTIC TOOL FOR *TRYPANOSOMA BRUCEI RHODESIENSE*

Christopher Kariuki, John Kagira, Simon Karanja, Naomi Maina and Maina Ngotho

Institute of Primate Research, Karen, Nairobi, Kenya; JKUAT, Kenya

Human African trypanosomiasis (HAT) is a vector borne parasitic disease. It is considered a neglected re-emerging disease of public health importance affecting mainly the poor in remote rural African communities. The diagnosis, particularly the stage determination of the disease is critical in the choice of therapeutic intervention. There is need for improvement of diagnostic methods as parasite detection remains insufficiently sensitive and cumbersome. Up to now, a simple serological field applicable diagnostic test for T. b. rhodesiense infection is yet to be implemented. Nanobody® (miniature antibody) technologies are a recent discovery in the field of disease diagnosis. Derived from an *in vivo* matured camelid immune cell complementary DNA (cDNA), nanobodies have high potential for diagnostic applications in HAT. These recombinant miniature antibodies can be engineered against the conserved regions of variable surface coat antigens on the trypanosome and, conjugated to molecules to increase their sensitivity.

Poster 8:

COMPARISON OF NUCLEIC ACID SEQUENCE-BASED AMPLIFICATION AND LAMP FOR DIAGNOSIS OF SLEEPING SICKNESS

Claire M. Mugasa[1], Enock Matovu[1], Diana Katiti[1], Alex Boobo[1], George W. Lubega[1], Henk D.F.H. Schallig[2,]

[1]School of Biosecurity, Biotechnical and Laboratory Sciences, COVAB, Makerere University Kampala, Uganda., [2]Koninklijk Instituut voor de Tropen (KIT)/Royal Tropical Institute, KIT Biomedical Research, Amsterdam, The Netherlands.

Molecular diagnostic tests are paramount especially in diagnosis of *Trypanosoma brucei gambiense* infections where the parasitaemia is usually very low. In this study we compare two simplified molecular tests for the diagnosis; for nucleic acid sequence-based amplification (NASBA) and loop- mediated isothermal amplification (LAMP) of clinical samples from patients and serological suspects of *T. b. gambiense* infection in Uganda. A total of 116 samples were analyzed and the diagnostic sensitivity and specificity of NASBA was 90.6% (95%CI; 80.7-96%) and 100% (95%CI; 94.9-100%), while the sensitivity and specificity for LAMP as compared to microscopy as the reference test was 79.7% (95%CI; 68.2-87.9%) and 100% (95%CI; 91.6-100%) respectively. Both index test showed a highly significant statistical difference in accuracy when compared to microscopy (P=0.0001). Of the suspect samples tested, 4 (13.3%) and 18 (60%) of the samples tested positive with NASBA and LAMP respectively, and 3 of these samples gave a positive result in both NASBA and LAMP. LAMP and NASBA-OC showed comparable diagnostic accuracy when used to diagnose HAT. Larger studies are recommended to explicate the function of these tests in detecting CATT suspect samples and follow-up of such to parasitological conversion of the suspects.

Poster 9:

PAPER-BASED COLORIMETRIC TEST STRIPS

Clémence Sicard,1 Madiha F. Khan,2 Michael A. Brook,1,2 and John D. Brennan1

1 Department of Chemistry and Chemical Biology, McMaster University, 1280 Main Street West, Hamilton, ON, L8S 4M1.
2Department of Biomedical Engineering, McMaster University, 1280 Main Street West, Hamilton, ON, L8S 4M1.

Our research group has developed simple, low-cost, paper-based colorimetric test strips for the detection of water and food contaminants, such as organophosphate pesticides,1 heavy metals,2 and coliforms.3 The test strip utilizes sol-gel based bio-immobilization methods in conjunction with ink-jet printing for the deposition of various reagents onto paper to produce paper-assays.4 The ink-jet printing can be performed with a piezo ink-jet printer (Fujifilm Dimatix DMP-2800) or a thermal ink-jet printer (Canon Pixma MP280) and when required a hydrophobic barrier can be created with a wax printer.5 We will demonstrate the use and fabrication of paper test strips on site. Preliminary results from the 2012 field tests in Kisumu, Kenya will also be discussed.

(1) Hossain et al, Anal. Chem. 2009, 81, 9055; (2) Hossain et al, Anal. Chem. 2011, 83, 8772; (3) Hossain et al, Anal. Bioanal. Chem. 2012, 403, 1567; (4) Hossain et al, Anal. Chem. 2009, 81,5474; (5) Carrilho et al, Anal. Chem. 2009, 81, 7091

Poster 10:

CHALLENGES IN ESTABLISHING POINT OF CARE TESTING IN A DEVELOPING COUNTRY; THE AGA KHAN UNIVERSITY HOSPITAL EXPERIENCE

Daniel Maina
Aga Khan University Hospital, Nairobi

The AKU hospital committee is tasked with the responsibility of overseeing the formulation and implementation of Point of Care Testing policy within the hospital. Amongst the challenges faced by the committee are issues such as training the end-users on quality assurance, method validation, and the fact that we don't have national guidelines or a regulatory framework to regulate POCT and/or equipment used across various institutions.

Poster 11:

IL-6 AS A MARKER FOR *TRYPANOSOMA BRUCEI RHODESIENSE* LATE STAGE DISEASE IN EXPERIMENTALLY INFECTED VERVET MONKEY

D.N. Maranga[a], J.M. Kagira [c] ,C.K. Kinyanjui[c], S.M. Karanja[a], [b], M. Ngotho [c], & N.W.N. Maina [a]

[a] *Jomo Kenyatta University of Agriculture and Technology (JKUAT), Biochemistry department, Nairobi, Kenya*
[b] *Institute of Tropical Medicine and Infectious Diseases, JKUAT, Nairobi, Kenya*
[c] *Institute of Primate Research, Karen, Nairobi, Kenya*

Background Human African trypanosomiasis (HAT) or sleeping sickness is a tropical and infectious disease that leads to a complex neuropsychiatric syndrome. It is characterized by two stages, that is, early hemolymphatic stage and late meningoencephalitic stage, primarily based on the detection of white blood cells and/or trypanosomes in the cerebrospinal fluid. The validity of this criterion is, however, debated, and novel laboratory biomarkers are required for accurate staging and subsequent treatment. IL-6 has been shown to play a significant role in neuropathogenesis and as a blood-brain barrier modulator with potential as a parameter for HAT stage determination. Methods Seven adult vervet monkeys (Chlorocebus aethiops) were utilized in this study. Four were experimentally infected with Trypanosoma brucei rhodesiense and treated sub-curatively at 28 days post-infection. Three control animals remained uninfected. Cerebrospinal fluid (CSF) and blood samples were obtained at weekly intervals for 56 days. Sera harvested from blood were used to quantify IL-6 by flow cytometry. Total protein and albumin were quantified in the sera and CSF samples using colorimetric techniques. Results: The serum IL-6 was up-regulated in the transitional stage of infection while CSF IL-6 was up-regulated in the late stage coinciding with relapse parasitaemia. Serum albumin showed a significant decrease ($P < 0.05$) upon infection only returning to pre-infection levels on sub-curative treatment. Serum total protein significantly increased ($P < 0.05$) in early stage of infection and only returned to pre-infection levels after sub-curative treatment. Cerebrospinal fluid total protein levels showed a significant increase ($P < 0.05$) in late stage of infection.

Poster 12:

DIAGNOSTICS PRACTICING VETERINARIANS

Dennis Omondi

University of Nairobi

As a clinician correct diagnosis defines the management outcome of the patient and being in a third world country, Kenya it is almost everyday that one has limited resources to aid in the diagnosis. This has prompted use of simple diagnostic methods which most have been outdated and I would really love this opportunity to get updated and equipped with these skills for better management of my patients.

Poster 13:

A COMPARISON OF DIAGNOSTIC METHODS AGAINST PCR FOR THE DETECTION OF *S. MANSONI* AMONG SCHOOL CHILDREN IN WESTERN KENYA

Elizabeth A. Ochola[1], Daniel O. Matete[1], Karen T. Foo[2], Pauline N. M. Mwinzi[1], John M. Williamson[1], Susan P. Montgomery[2], Eric Brienen[3] , Jaco J. Verweij[3], L. VanLieshout[3], W. Evan Secor[2], and Diana M. S. Karanja[1].

1 Center for Global Health Research, Kenya Medical Research Institute, P.O. Box 1578-40100, Kisumu, Kenya;
2 Division of Parasitic Diseases and Malaria, Centers for Disease control and Prevention,1600 Clifton Rd, N. E.,Mailstop D-65, Atlanta,
3 Leiden University Medical Center, Department of Parasitology (ZoneP4-P) Albinusdreef 2 2333 ZA Leiden, The Netherlands

The most widely used tools for diagnosis of S. mansoni, stool examination and serologic detection, are limited by low sensitivity or inability to distinguish between current from former infections, respectively. As a result, there is not an accepted “gold standard” for S. mansoni diagnosis for evaluation of new diagnostic tools. However, recent development of a semi-quantitative PCR that detects schistosome DNA stool provides a tool with increased sensitivity and specificity for this purpose. We utilized the PCR method to help evaluate two versions of a point of contact (POC) test designed to detect circulating cathodic antigen (CCA) in urine of people infected with schistosomes. 1898 school children aged between 8-12 years from Rarieda District of western Kenya took part in the study. Three stool and three urine samples were collected on consecutive days for testing by Kato-Katz and the CCA cassettes. A portion of the first day’s stool was preserved in ethanol and subsequently tested for presence of schistosome DNA by PCR.In addition, serum from a single blood sample was tested by ELISA for anti-schistosome IgG. Children who were infected with S. mansoni were treated using praziquantel. Results: Compared to PCR, the initial version of the POC urine test (CCA-M) on any of the 3 days had the highest sensitivity at 92.11%, followed by Kato=-Katz at 70.44%. The alternate version of the POC test (CCA-A) and ELISA had sensitivities of 52.56% and 50.16%, respectively. This results show that PCR is a promising gold standard assay that can be used when evaluating the performance of different tests used for the diagnosis of S.mansoni infection present in Schistosomiasis endemic areas.

Poster 14:

EFFECTS OF UNREGULATED USE OF GLUCOMETER ON MANAGEMENT OF PATIENTS

Erick Atema

Bungoma District Hospital

Glucometer is a patient monitoring and management tool used in measuring blood glucose level. The medical device is commonly used to measure blood glucose level in diabetic patient and on patients on medication: it producers within 6o seconds in addition it uses very little blood from finger tips. Fortunately or unfortunately the device has been manufactured by different companies all over the world this has been a benefit to consumer and also a curse: as result of mass production of glucometers there has been significant variation on results among the medical device. To contain this situation food and drug administration has certified glucometers to be used in blood glucose measurement: however this initiative is still to be adapted by most countries. Glucose level variation more than 1.0mmol/L or more than 10% significantly changes the direction of management of the patient. These changes are critical while handling hypoglycemic or hyperglycemic patient A higher variation will mislead clinicians in managing the patients: as result death may occur due to hypoglycemia ,most diabetic patients will willingly buy this device unaware of its accuracy to produce accurate blood glucose measurement, this posses a great challenges in patient managing himself and herself alone

Poster 15:

A STUDY ON THE INCIDENCE OF TSETSE FLY IN RELATION TO TRYPANOSOMOSIS PREVALENCE AND ITS IMPACT ON LIVESTOCK PRODUCTION IN MPANDA DISTRICT – TANZANIA

Lyaruu, Eugen

Tsetse & Trypanosomiasis Research Institute, Tanazania
Livestock Research officer, Tsetse & Trypanosomiasis Research Institute (TTRI) , P. Box 1026 TANGA, TANZANIA mobile 0786-067802
Email: euglyaruu@yahoo.com

Tsetse flies (Genus Glossina) are the primary vector of trypanosomosis and the only vector capable of transmitting trypanosomes cyclically. This vector is the causative agent for African animal trypanosomosis (Nagana) and Human African trypanosomosis (Sleeping sickness). Tanzania has an area of approximately 937,000 square kilometers and about 60% of the land is infested with seven tsetse species. Trypanosomosis (Nagana) is considered to be one of the most important disease of livestock and is estimated that some 4.4 million livestock are at risk (FAO, 2000), the disease reduces the productivity directly as a consequence of mortality and morbidity and indirectly through its impact on land use and rural development where as farmers normally tends to avoid tsetse infested areas. Mpanda district has 321,000 hectares of land infested by tsetse fly, the areas infested by tsetse fly including the following villages Ugalla, Mnyamasi, Karema, Mpimbwe and Ihumbe. The number of livestock in this district are 219,779(Cattle), 105,063(Goat) and 70,976(Sheep). The presence of tsetse and trypanosomosis in this district cause a big constraint to livestock development through its effect on livestock distribution as the areas which are tsetse infested have reliable source of water and good pastures but livestock keepers tends to avoid this areas and concentrated in small area which is tsetse free and thus cause land degradation due to overgrazing as resulting to environment destruction leading to desertification and poor nutrition to livestock which influence low livestock production items of milk and poor quality meat. Ugalla village is known as endemic foci for Human African trypanosomosis (HAT) so in this village there is a committee for tsetse control and currently deployment of impregnated targets is going on, this project is aiming to integrate different techniques for tsetse and trypanosomosis control. The vision of this project is to reduce trypanosomosis prevalence in livestock by 90% this can be achieved through control of insect vector (tsetse fly).

Poster 16:

BABOON (*PAPIO ANUBIS*) AS A MODEL FOR DEVELOPING EFFECTIVE DIAGNOSTIC TOOLS FOR PLACENTAL MALARIA IN HUMANS

Onditi F[1,2], Mustafa B[3], Omwandho C[2],, Ozwara H[1], Farah IO[1], Moore J[3]

[1]Institute of Primate Research, National Museums of Kenya, P.O. Box 24481– 00502, Karen, Kenya, [2]University of Nairobi, P.O. Box 30197–00100, Nairobi, Kenya, 3Kenyatta University, P.O.Box 43844-00100, Nairobi, Kenya.4Center for Tropical and Emerging Global Diseases & Department of Infectious Diseases N330C Paul D. Coverdell Center 500 DW Brooks Drive University of Georgia Athens, GA 30602

Malaria in pregnancy is a public health problem in sub-Saharan Africa and represents enormous diagnostic challenges such as nonspecific clinical representation of malaria, lack of effective diagnostic facilities and personnel, and absence of regulatory standards for diagnostic tests. Reproducible animal models are required to overcome these challenges. The human-like structure of the baboon placenta and the cyto-adherent property of Plasmodium knowlesi justify the baboon-P. knowlesi model. In our approach, pregnant and non-pregnant baboons were experimentally infected with P. knowlesi H strain parasites (1x106). They were monitored for parasitaemia, clinical symptoms (from day 2 post infection) and histopathology. Our findings show that parasitaemia levels of the non-pregnant infected (NPI) group was higher (4%) than the pregnant infected (PI) group (3%). The placental parasitaemia was on average over 19 fold higher than the peripheral parasitaemia in the same animal. Histopathological findings confirmed placental parasitaemia and sequestration. Findings in the baboon-P. knowlesi model correlate well with findings observed in humans. Consequently, the baboon- P. knowlesi model of malaria is a promising model of choice that can be used in the development of an effective diagnostic tool for placental malaria.

Poster 17:

DIAGNOSTICS FOR ZOONOSES

Fred Maloba

INSTITUTE OF PRIMATE RESEARCH

Being a scientist and veterinarian I would be interested to learn quick and low cost diagnostics which are essential especially in the African continent. This because of the low high poverty level which limits many people to access good health care for themselves and for their livestock

Poster 18:

DIAGNOSTICS EVALUATION AND PERFORMANCE LABORATORY FOR OPERATIONAL YARDSTICKS

Tuan Nguyen, Tom Slezak, Crystal Jaing, Larry Dugan, Reg Beer and George R. Farquar

Lawrence Livermore National Laboratory

Lawrence Livermore National Laboratory (LLNL) is currently developing a new laboratory capability specifically designed to evaluate new and developing diagnostic platforms. The Diagnostics, Evaluation and Performance Laboratory for Operational Yardsticks (DEPLOY) is housed in a set of existing laboratories that have the capability to evaluate the engineering as well as the biology of diagnostic tests. LLNL's capabilities for detector testing and evaluation come from over 20 years of research, development, testing, evaluation and fielding in biological detection and diagnostic testing. The presentation will also highlight three of our ongoing projects in diagnostics (DNA-Microarrays, LAMP and fast PCR).
LLNL has developed a DNA Microarray that focuses on vertebrate-infecting pathogens (as opposed to just human-infecting) because effective biosurveillance in Kenya or other developing nations must have the capability to establish baselines and detect deviations for not only humans but also the other hosts, vectors, and reservoirs.
LAMP isothermal amplification has shown promise in limited resource settings due to its simplicity. We have designed, and tested a colorimetric, disposable Point-of-Need (PON) molecular diagnostic that accomplishes sample preparation, isothermal nucleic acid amplification and detection in a single tube in about 1 hour.
PCR is the State-of-the-art for molecular diagnostics and detection because of its unmatched specificity and sensitivity. Existing PCR instruments require ~30 minutes (typical of fast systems) to perform their assays. We achieved 30 cycles of amplification in two minutes and eighteen seconds.

This work performed under the auspices of the U.S. Department of Energy by Lawrence Livermore National Laboratory under Contract DE-AC52-07NA27344.

Poster 19:

TUBERCULOSIS IN CAMELS AND HUMANS IN PASTORAL REGIONS OF ETHIOPIA

Gizachew Bayleyegn

Haramaya University

A cross sectional study was conducted on 906 apparently healthy slaughtered camels and on 120 suspected humans in Ethiopia to investigate the pathology of camel tuberculosis (TB) and characterize its causative agents as well as to assess public health importance of the disease. The prevalence of camel TB was 10.04% (91/906) on the basis of pathology and it was significantly higher in females (x2 = 4.789; P = 0.029). Mycobacterial growth was observed in 34% (31/91) of camels with grossly suspicious TB lesions. Upon further molecular characterization using multiplex PCR, 68% (21/31) of the colonies showed a positive signal for the genus Mycobacterium, of which two were confirmed Mycobacterium bovis by RD4 deletion typing. Further characterization of the two M. bovis at strains level revealed that one of the strains was SB0133 while the other strain was new and had not been reported to the M. bovis database prior to this study. Hence, it has now been reported to the database, and designated as SB1953. On the other hand, 6 of the 8 human isolates subjected to RD9 deletion typing were confirmed to be M. tuberculosis. In conclusion, further genotyping of isolates is useful towards the efforts made to control TB.

Poster 20:

HAEMATOLOGICAL CHANGES IN SHEEP (*OVIS ARIES*) INFECTED WITH HUMAN INFECTIVE *TRYPANOSOMA BRUCEI RHODESIENSE*

H.K.M. Mavura[1], R.M.Ngure[1] , G.Murilla[2] , M. Wachira[3]

[1]Egerton University, 2 KARI-TRC, 3 Laikipia University College

The haematological parameters of sheep (*Ovis aries*) an experimental model of human African trypanosomiasis were determined. Ten sheep were used ;eight animals were infected with 1× 104 *Trypanosoma brucei rhodesiense* while two animals were not infected and hence saved as control .Clinical evaluation was done daily while weekly animals of both groups were bled and blood samples examined for packed cell volume (PCV), haemoglobin (Hb), red blood cell count (RBC), platelet counts, Total white blood cell counts and erythrocyte indexes of mean corpuscular volume (MCV), mean corpuscular haemoglobin (MCH), mean corpuscular haemoglobin concentration and (MCHC) . Clinical evaluation of the disease in the sheep corresponded closely to that described in human patients. Infected sheep showed an increase in body temperature, MCH and MCHC. However the levels of PCV, Hb, MCV and RBC counts declined drastically on infection. All parameters however recovered to control after treatment. Analysis of data revealed significant changes in all these parameters and erythrocyte indexes in infected sheep when compared with controls. From the changes observed in this study, we can conclude that macrocytic anemia was the major feature of the infected sheep.

Poster 21:

IMPORTANCE OF RAPID AND SENSITIVE DIAGNOSTIC IN ACCURATE ESTIMATION OF HUMAN AFRICAN TRYPANOSOMIASIS MAGNITUDE IN ENDEMIC AREAS IN TANZANIA

Malele, I.I.[1], H. Nyingilili[1], E. Lyaruu[1] & W. Kitwika[2]

[1]Tsetse & Trypanosomiasis Research Institute (TTRI), Box 1026 Tanga, TANZANIA
[2]Tsetse & Typanosomiasis Research and Control Centre, Box 1380, Kigoma, Tanzania

Sleeping sickness is one of the neglected tropical diseases. More than 60% of the total cases of Human African trypanosomiasis (HAT) reported locally originate from western Tanzania. Cases from Serengeti Ecosystem are reported mostly among tourists. HAT cases, are directly related to the prevalence of human infective trypanosomes in the vectors or host animals. Traditional detection microscopically have short comings as the method doesn't discriminate between human and animal disease causative parasites and is less sensitive. Reliance on such data, tend to underestimate the actual prevalence and thus the risks of HAT. Molecular diagnostic tools were incorporated in the analysis of vector samples collected from 2009 to 2011. Trapped tsetse were dissected and infected and uninfected organs were further analysed by SRA LAMP. The infection rate in tsetse salivary glands was 1/36 microscopically and 2/36 by SRA LAMP in 2009. In 2010 and 2011, microscopic infection was 9/36 and all were positive by SRA LAMP; and 11/50 positive microscopically but 7/50 positive by SRA LAMP respectively. The result by SRA LAMP confirms the presence of human infective trypanosomes circulating in the area and calls for the integration of rapid and sensitive diagnostic techniques in accurate estimation of the magnitude of HAT causative parasites and the risks posed by HAT. Rapid and diagnostic techniques are needed to revolutionize diagnostic of HAT in poor resource settings in rural areas.

Poster 22:

SAFETY EVALUATION OF TRADITIONALLY USED ANTIMALARIAL PLANTS

J.M.Nguta, J.M.Mbaria, D.W.Gakuya, P.K.Gathumbi, J.D.Kabasa, S.G.Kiama

University of Nairobi, Nairobi, Kenya

Over 250 million cases of malaria are reported each year, 1 million of which result in death. Currently, no drugs are universally successful in treating malaria due to widespread drug resistance; new antimalarials from natural products of plant biodiversity are desperately needed. However few data are available on their safety, despite the fact that validation of traditional practices could lead to innovative strategies in malaria control. Out of the 45 organic extracts screened for activity against *Artemia salina* larvae, 23 (51%) of the crude extracts demonstrated activity at or below 100 µg/ml, and were categorized as having strong cytotoxic activity, whereas 20% (9) of the aqueous extracts demonstrated activity at or below 100 µg/ml and were considered to have strong activity against *Artemia salina* larvae. In the current study, 95.5% of all the screened organic extracts and 76% of the investigated aqueous extracts demonstrated LC50 values <1000 µg/ml, indicating that these plants could not make safe antimalarial treatments. This calls for dose adjustment amongst the community using the plant extracts for the treatment of malaria and chemical investigation for isolation of bioactive compounds responsible for the observed toxicity

Poster 23:

SIMPLE ANALYTICAL DEVICES SENSITIVE TO THE SIZE, SHAPE AND DEFORMABILITY OF CELLS

Jason P. Beech, Stefan H. Holm, Karl Adolfsson and Jonas O. Tegenfeldt

Lund University

While size has been widely used as a parameter in cellular separations, shape and deformability remain largely untapped sources of specificity in preparative and analytical microfluidic devices. Devices that are relatively simple to fabricate and use but that are very sensitive to the size, shape and deformability of cells could be used for a wide range of analytical and preparative processes without recourse to expensive cytometers. Furthermore, labelling of cells is not necessary, helping to keep simplicity and cost at a minimum.

Our method is based on Deterministic Lateral Displacement1, a mechanism which causes particles to follow trajectories through arrays of micrometre-sized posts based on size, shape and deformability. We have previously shown how morphological differences can be used to isolate parasites from blood2, a method we hope could find utility in the fight against Human African Trypanosomiasis. We have also shown how the deformability of red blood cells can be measured and used as a separation parameter3, a parameter that is greatly affected by infection by the Malaria parasite.

The primary questions that remain to be answered, if these devices are to become useful for Point of Care diagnostics are: How simple yet reliable can the fluidic control be? Do we really need large expensive microscopes to see the results of our analysis? And, can we actually deal with real samples taken in the field?

1. L. R. Huang, E. C. Cox, R. H. Austin and J. C. Sturm, *Science*, 2004, **304**, 987-990.
2. S. H. Holm, J. P. Beech, M. P. Barrett and J. O. Tegenfeldt, *Lab on a Chip*, 2011, **11**, 1326-1332.
3. J. P. Beech, S. H. Holm, K. Adolfsson and J. O. Tegenfeldt, *Lab on a Chip*, 2012, **12**, 1048-1051.

Poster 24:

EFFECTS OF HERBAL MEDICINES USED BY HERBAL PRACTITIONERS ON THE REPRODUCTIVE PROCESS

Jemimma Oduma

University of Nairobi

My area of expertise is Physiology of Reproduction. I am currently doing a lot of research through postgraduate students on effects of herbal medicines used by herbal practitioners on the reproductive process. The aim is to prove or disprove the efficacy of such compounds in laboratory animals with a view to moving them forward to formal remedies. This workshop, Point of Care Diagnostic Workshop will be very appropriate especially on clinical aspects of my research.

Poster 25:

TOWARDS THE DEVELOPMENT OF AN IMPROVED DIAGNOSTIC TEST FOR CONTAGIOUS BOVINE PLEUROPNEUMONIA

Nimmo Gicheru, Anne Liljander and Joerg Jores

International Livestock Research Institute, PO Box 30709, 00100 Nairobi, Kenya

Contagious bovine pleuropneumonia (CBPP) is an important cattle disease, which is present in many countries of sub-Saharan Africa. It is caused by Mycoplasma mycoides subsp. mycoides. CBPP greatly impacts livestock-dependent communities by restricting market access and reduced food supply. The current diagnosis of CBPP relies on tests that have limited sensitivity and are therefore only useful at herd level.

The aim of the project was to identify and compare immunogenic Mycoplasma proteins for the development of improved diagnostic assays for CBPP.

Immunogenic Mycoplasma proteins have been identified using two-dimentional electrophoresis combined with immunoblot, phase display libraries, as well as Luminex assay. Synthetic versions of Mycoplasma genes have been cloned, expressed and purified and subsequently tested using ELISA. The proteins were screened for their potential as diagnostic antigens using more than 100 well-defined experimental and field sera. Additionally, the presence of the corresponding genes has been verified in a large collection of M. mycoides subsp. mycoides strains.

Poster 26:

POTENTIAL UTILIZATION OF NATURAL PRODUCTS IN THE TREATMENT OF SLEEPING SICKNESS

Johnson Nasimolo

University of Nairobi

I am working on the second stage of trypanosomiasis using a mice model, and potential utilization of natural products in the disease. Diagnostic markers are important in determining cure in the mice model.

Poster 27:

GENOTYPING OF *TRYPANOSOMA CONGOLENSE* IN CATTLE AND GLOSSINA PALLIDIPES (TSETSE FLIES) IN SHIMBA HILLS AREA, KENYA.

[1,3] Muthinja J., [3] Muigai A., [1,2] Ciosi M., [2]Turner M., [1]Masiga D

[1]International Centre of Insect Physiology and Ecology. P. O. Box 30772-00100 Nairobi, Kenya., [2]University of Glasgow. Glasgow, G12 8QQ, Scotland. [3]Jomo Kenyatta university of Agriculture and technology. P.O. Box 62,000 - 00200 Nairobi, Kenya

Trypanosoma congolense belongs to the subgenus Nannomonas and is the lead causative agent for African Animal Trypanosomiasis (AAT). T. congolense infections cause losses worth 4.5 billion dollars annually to livestock dependent economies in Sub-Saharan Africa. Thus, trypanocidal drugs are in use. However, parasites resistant to these trypanocides have emerged making the treatment of AAT difficult. Parasite resistance to trypanocides, virulence and infectivity, are variable phenotypes revealed during infection. Consequently genotyping provides a useful link between the genetic component and phenotypic manifestation. In this study the ecology, identity and genetics of trypanosomes circulating between bovine hosts and *G. pallidipes* in areas surrounding the Shimba hills game reserve, Kenya was considered. Six out of the eleven trypanosome species that are detectable by PCR were found to be present in this area, making the percentage level of diversity 54%. This level of diversity was observed for both cattle hosts and *G. pallidipes*. This finding will greatly influence future sampling studies in this area, whereby either the vector or host will be used to source trypanosomes. Microsatellite genotyping revealed six diverse genotypes of *T. congolense* subtype savannah. Interestingly two out of the six genotypes were found in a single fly proboscis.

Poster 28:

DEVELOPMENT OF SENSING PLATFORM FOR TB-SPECIFIC ANTIBODIES USING PAPER-BASED BACTERIA CULTURES AND ENGINEERED BACTERIOPHAGE

Katrina Tjhung (1), Maribel Funes (2), Simon Ng (1), Wadim Matochko (1), Emanuel Carrilho (2), Ratmir Derda (1)

(1) Dept. of Chemistry, University of Alberta
(2) Institute of Chemistry of Sao Carlos, University of Sao Paulo, Brazil

Incidences of diseases such as HIV, malaria, and tuberculosis (TB) are devastating in developing countries. Poor diagnosis and treatment of diseases in these particular regions is exacerbated due to the lack of resources for purchase or production of diagnostic devices.

To address this issue, we aim to develop a point-of-care diagnostic tool using cost efficient materials that is easy to store and easy to analyze, without depending on costly equipment. Our tool uses engineered bacteriophage to detect biomarkers of disease. The advantages of using bacteriophage as opposed to widely used antibody diagnostic assays are that bacteriophage are simply and easily produced from relatively inexpensive materials, can be stored at ambient conditions, and by the nature of bacteriophage reproduction, produces a strong signal from a single detection event.

The principles of this technique involve taking advantage of the infectivity of phage to produce a signal. The coat protein pIII allows the phage to be infective when intact. However, when pIII is cleaved into two halves, the phage has no infectivity. We aim to cleave and modify pIII such that it will come together again only in the presence of the target biomarker: a circulating antibody against TB antigens. Hence, bacteriophage will infect E. coli to produce a signal only in the presence of model antibody.

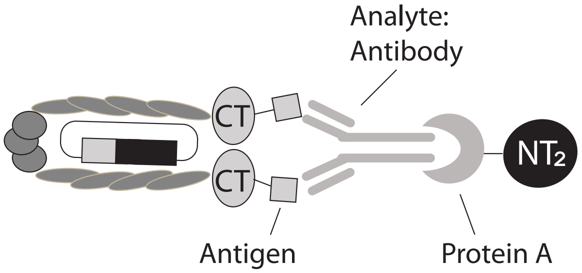

Fig. 1 – Incorporating antibody into bacteriophage infection mechanism. Modified Phage is infective.

Poster 29:

POPULATION GENETICS OF *PLASMODIUM FALCIPARUM* AND THE HOST

Elijah Githui

National Museums of Kenya

One of the research work focus on population genetics of Plasmodium falciparum and the host. Our study focuses on Erythrocyte Binding protein (EBL-1) and the host Glycophorin B. We are analyzing single nucleotide polymorphisms within study of defining the direction of microevolution of these proteins. We currently are interested in expanding the work to demographic genome wide analysis of the gene isolates from regions within Kenya in relation to global signatures at CEPH Hapmap.

Poster 30:

THE IMPACT ON THE LONGEVITY OF *ANOPHELES GAMBIAE* INFECTED BY DRUG RESISTANT *PLASMODIUM FALCIPARUM*

Kevin Kamanyi

Egerton University, Kenya

The main vector for Plasmodium falciparum in sub-saharan Africa is *Anopheles gambiae*. *P. falciparum* is the most virulent human malaria parasite. The difficulties in combating malaria are partly due to rapid resistance emergence by the parasites to the available antimalarial drugs. However, the impact of the drug resistance on the development of *Anopheles gambiae* is unknown. In this study, the impact on the infection efficiency of the drug resistant parasites to An. gambiae and the vector survival rate after successful infected blood meal will be studied. Two hundred laboratory adapted An. gambiae s.s. mosquitoes will be fed on gametocytes of cultured Sulfadoxine pyrimethamine (SP) resistant laboratory strain of *P. falciparum* (Dd2) via an artificial membrane feeding system. Fully engorged mosquitoes will be maintained on 10% glucose solution in an insectary at relative humidity of 75-80%, and a temperature of 25-27oC and mortality rate monitored twice daily. To estimate the parasite load on the dead mosquitoes, the mosquitoes will be dissected and their head, thorax and abdomen analyzed using circumsporozoite protein ELISA. These data will be compared with those obtained from mosquitoes fed on a SP sensitive laboratory P. falciparum (M24) strain. It is expected that the longevity of the mosquitoes infected with drug resistant strain will be significantly different compared to the mosquitoes infected with the sensitive P. falciparum. This will be an indication that drug resistant P. falciparum strains also changes in virulence in addition to its mutation associated with drug pressure evasion. This study will shed light on P. falciparum virulence vis-a-vis drug resistance. Understanding how the parasite resistance impacts on the longevity of the vector may also aid in malaria transmission control strategies. In addition, a masters degree and a publication of this work on a peer reviewed journal will be achieved.

Poster 31:

DIAGNOSTICS AT MOI UNIVERSITY SCHOOL OF MEDICINE

Kirtika Patel

Moi University, Kenya

I am a lecturer and investigator in Immunology in the School of Medicine at Moi University, Eldoret, Kenya. I have been involved in initiating the setting of Immunohistochemistry (IHC) Laboratory, a first of its kind in Western Kenya. Have worked on mutational and risk factor analysis for esophageal cancer. Currently, working on evaluation of hormone-receptor status and growth factor receptor expression as predictive factors for breast cancer prognosis, using FACS and IHC for diagnosing lymphomas and Kaposis Sarcoma, PCR for HPV detection in cervical cancer and molecular analysis of ethnic variation and molecular fingerprint in Wilms' tumor. Other goal is to establish a tissue repository. I am hoping to develop easier ways for diagnosing cancer in a limited resource setting like ours. This workshop will expose me to various research ideas and help in setting up of cheaper and easier way to test for cancer and thus help in delivering excellent point of care.

Poster 32:

ESTABLISHMENT OF DIAGNOSTIC TECHNOLOGIES FOR INCREASED ANIMAL PRODUCTION IN UGANDA

[1]Lonzy Ojok, [1]Okuni Julius B, [2]William Olaho-Mukani

[1]Makerere University, [2]Ministry of Agriculture Animal Industries and Fisheries.

Our research in on African swine fever, a highly fatal devastating disease of pigs. Trying to find out the most common strains present in the country and develop antigen and antibody based diagnostic tools for the disease. Specific viral protein and recombinant protein will be produced and used in the development of the diagnostic technologies. The title of the research work is "Establishment of diagnostic technologies for increased animal production in Uganda.

Poster 33:

MALARIA DIAGNOSIS: NEED TO TRACK THE CHANGING FACE OF MALARIA TRANSMISSION

Lucy Ochola[1], Thomas Kariuki[1], Bernhards Ogutu[2]

[1]Institute of Primate Research, Nairobi, Kenya, [2]Centre for Clinical Research, Kenya Medical Research Institute

Increased investment in malaria research and control leading to improved distribution of insecticide-treated bednets and introduction of artemesinin derivatives in the last two decades has resulted in an overall reduction in the incidence of malaria disease. This has been coupled with a reduction in transmission levels making malaria elimination a possibility. However, complete control and eradication cannot be achieved within a framework of poor diagnostic techniques and policies that tend to ignore low grade parasitaemia especially in asymptomatic carriers that provide a pool for transmission of malaria. The current techniques for malaria diagnosis include microscopic examination of peripheral blood films, antigen detection using rapid diagnostic tests (RDTs) and molecular based methods. RDTs and microscopy may not be reliable especially when parasitaemia fall below 5-10parasites/µl, while molecular methods have a sensitivity and specificity of 100% at 1 parasite/µl but are yet to be developed for point of care use. Microscopy is subjective and heavily relies on the proficiency of the user. Furthermore, there is evidence that RDTs may not be very sensitive especially in varying transmission intensities and ecological settings. We discuss the importance of diagnostic techniques in the fight to eradicate and control malaria and foreseen challenges and possible solutions.

Poster 34:

EPIDEMIOLOGY OF TOXOPLASMOSIS AND IDENTIFICATION OF PARASITE GENOTYPES INFECTING HUMANS IN THIKA DISTRICT, KENYA

Ichagichu M., Kagira JM, Karanja SM., Maina N., Ngotho JM

INSTITUTE OF PRIMATE RESEARCH, JKUAT

My PhD research involves studies on prevalence of toxoplasmosis. Toxoplasmosis affects all warm-blooded hosts, including humans. Toxoplasmosis is a leading cause of infectious reproductive failure in humans and animals, and severe disease in congenitally infected children and immunocompromised people. Despite its importance, information on the occurrence and control of this zoonosis in Kenya is limited. The project aims to determine burden of toxoplasmosis and identify *T. gondii* genotypes infecting humans in Thika District, Kenya. Study population being surveyed includes livestock keepers, slaughterhouse workers, pregnant women, HIV-positive patients and individuals with visual and mental handicaps. The tests being used in the study includes ELISA, PCR, LAMP and sequencing.

Poster 35:

VALIDATION OF MOLECULAR DIAGNOSTIC TECHNIQUES FOR AFRICAN ANIMAL TRYPANOSOMIASIS (AAT) IN TANZANIA

Mechtilda Byamungu[1], Adama Diallo[2], Lelenta Mamadou[3], Debastian W. Chenyambuga[4]

1. TSETSE & TRYPANOSOMIASIS RESEARCH INSTITUTE, Tanzania
2. Dr. Adama Diallo: Animal Production Unit, Seibersdorf Agriculture and Biotechnology Laboratories, Austria
3. Dr. Lelenta Mamadou; Animal Production Unit, Seibersdorf Agriculture and Biotechnology Laboratories, Austria
4. Dr. Sebastian W. Chenyambuga: Sokoine University of Agriculture Morogoro, Tanzania

Trypanosomiasis, is economically important cattle disease in Tanzania inflicting annual losses of 364 million US$ through lowered productivity. The disease problem is propagated by lack of sensitive techniques for diagnosis in most of the affected rural areas. The limitations of available parasitological and serological methods for diagnosis necessitate employment of sensitive and specific DNA-based diagnostic methods to facilitate epidemiological studies of trypanosomiasis. This research project aims at validating the developed primers in the previous study or design new ones for the detection of the three trypanosomes species (Trypanosoma congolense, T.vivax and T.b.brucei) causing African Animal Trypanosomiasis that are circulating in Tanzania. The validation will be done in phases by testing the sensitivity of the Loop Mediated Isothermal Amplification (LAMP) to monitor the efficacy of the treatment in experimental animals.
Animals (small ruminants and young cattle) will be cleared and infected then monitored using LAMP and parasitological technique for comparison for four and two week's respectively. Phase three will involve the validation of the technique at large scale. Blood samples will be collected randomly from livestock in the areas that are highly infested with Trypanosome species in Tanzania. After successful validation the technique will be made available to the veterinary department for implementation.

Poster 36:

DIAGNOSIS FOR AMOEBIASIS

Meshack Obonyo

Egerton University, Kenya

Entamoeba histolytica causes human amoebiasis and is among the most common parasitic infections worldwide infecting over 50 million people. Clinical manifestation is due to existence of two morphologically identical species but with different biochemical and genetic makeup. As such, it is of clinical importance to differentiate the two species (commensal and pathogenic), for treatment decision and public health interest. Infection occurs by ingestion of viable cysts from contaminated material such as water and food. Treatment is administered to all diagnosed cases of *E. histolytica* irrespective whether one has symptoms to avoid the risk of invasion, whereas cases found to involve only *E.dispar* should not be treated. Correct diagnosis, depends mainly on stool microscopy, which is slow and has low sensitivity and specificity thus cannot be relied upon to distinguish between the two due to its reliance on morphological characteristics. The current study was designed to differentiate *E.histolytica* and *E.dispar* by multiplex polymerase reaction in stool samples. The findings of this study are expected to highlight the degree of variation of sensitivity between the two methods and also determine the occurrence of the strains. This will form the basis for further research and management of the complex.

Poster 37:

DEVELOPMENT OF AN OLIVE BABOON MODEL FOR ALZHEIMER'S DISEASE

Michael Ndungu

INSTITUTE OF PRIMATE RESEARCH, Kenya

I am involved in the development of an Olive baboon model for Alzheimer's disease. The project entails occlusion of extra-cranial vessels to induce cerebral ischemia. Whole brain is subsequently harvested and pathological indices of Ad equivalence are assessed for with a view to mimic as closely as possible actual AD pathology. White matter lesions ar also examined to generate a template for vascular dementia staging. A major variable is the duration of occlusion and this has been shown to be a determinant in the extent and pattern of white matter pathology, glial cell proliferation and neuronal loss in the grey matter. The ultimate aim is to produce an animal model with the potential for testing and validating candidate therapeutic agents against degenerative diseases of the central nervous system.

Poster 38:

RAPID MALARIA DIAGNOSIS IN GHANA: IMPLEMENTING POLICIES, AND NAVIGATING TECHNOLOGY AT THE POINT OF CARE

Boadu N.Y.*[1], Ansong D.[2], Higginbottom G.[3], Einsiedel E.F.[4], Yanow S.K.[1,5]

[1]School of Public Health, 3-300 Edmonton Clinic Health Academy, 11405 87 Avenue, University of Alberta, T6G 1C9, Canada. Email: boadu@ualberta.ca
[2]Department of Child Health, School of Medical Sciences, Kwame Nkrumah University of Science and Technology, Kumasi, Ghana. Email: ansongd@gmail.com
[3]Faculty of Nursing, University of Alberta, 4-171 Edmonton Clinic Health Academy, 11405 87 Avenue, University of Alberta, T6G 1C9, Canada. Email: gina.higginbottom@ualberta.ca
[4]Department of Communication and Culture, University of Calgary, Alberta, T2N 1N4, Canada. Email: einsede@ucalgary.ca
[5]Research and Development – ProvLab, WMC 2B4.59, 8440 112 Street, Edmonton, AB, T6G 2J2
**Corresponding Author*

Background: Recently adapted World Health Organization (WHO) policies in Ghana recommend testing before treating all suspected malaria patients above five years of age. The original WHO guidelines refer to patients of all ages. Rapid diagnostic tests (RDTs) allow for quick and reliable diagnosis of malaria in peripheral facilities with limited laboratory capacity, without having to rely exclusively on clinical diagnosis. Implementing national health policies in limited-resource settings can be challenging for frontline health services workers. Such challenges may be amplified where implementation involves the uptake of new technology, such as RDTs. Studies reveal variable extents to which test results are followed for guiding fever case management across sub-Saharan Africa. Little is known about health workers' strategies for integrating RDT-use with fever case management in resource-constrained environments.
Objectives: 1) to examine national guideline adherence among health workers performing rapid malaria diagnosis; 2) to investigate health workers' strategies for point-of-care malaria testing with RDTs; and 3) to understand how health workers integrate policy with practice amidst limited resources.
Methodology/methods: A focused ethnography involving rural/peri-urban district health workers in direct observations, interviews and focus group discussions on rapid diagnostic testing for malaria between April and June 2012.
Results: This paper presents early findings on how health workers in this study integrate RDT-use and applied policies for rapid malaria testing in their facilities. Lessons drawn will be useful for informing optimization strategies in the application of RDTs and similar point-of-care diagnostic technologies in developing country health settings.

Poster 39:

BIOMARKERS FOR SLEEPING SICKNESS IN VERVET MONKEYS EXPERIMENTALLY INFECTED WITH *TRYPANOSOMA BRUCEI RHODESIENSE*

A. K. Gaithuma[1], S. M. Karanja[1], M. Ngotho[2], R. G. Maathai R[3], J. M. Kagira[2], Naomi. W.N. Maina[1]

[1] *Department of Biochemistry, Jomo Kenyatta University of Agriculture & Technology, P.O.Box 62000, Nairobi 00200, Kenya,* [2]*Institute of Primate Research, P.O. Box 24481 Karen 00502, Nairobi, Kenya,* [3]*Department of Biochemistry, University of Nairobi, P.O. Box 30596 - 00100, Nairobi, Kenya*

Human African trypanosomiasis is associated with metabolic changes and immunological changes which have not been well characterized. Four *Chlorocebus aethiops* were experimentally infected with *T. b. rhodesiense* and late stage disease induced at 28 days post infection (dpi). Ear prick blood for glucose determination and blood samples were obtained at weekly intervals for 63 days. Analysis was done using dry chemistry analysis. In early infection, there was a significant increase in creatine kinase, while during early and transitional stage of infection there was a significant decrease in blood glucose and high density lipoprotein (HDL) and an increase in triglyceride levels. In the advanced late stage, there was a significant increase in both total cholesterol and low density lipoprotein (LDL) levels. Further investigations should focus on levels of total cholesterol during the follow-up period in curatively treated vervet monkeys. Apart from their importance in disease staging, the changes in lipids levels may also affect the pharmacokinetics of some trypanocides.

Poster 40:

DIAGNOSTICS FOR ROTA VIRUS

Nicholas Kiulia, *INSTITUTE OF PRIMATE RESEARCH, Kenya*

Background: Group A rotaviruses are a major cause of acute gastroenteritis and severe dehydrating diarrhoea in children under five years of age. The World Health Organization has recommended that rotavirus vaccines be included in all national immunization programs as part of a strategy to control rotavirus-associated diarrhoeal diseases. Hospital - based surveillance of severe rotavirus diarrhoea is therefore crucial in monitoring the impact pre and post-vaccine introduction and also to document changes in genotype distribution. **Objectives**: To determine the molecular epidemiology of circulating rotavirus strains in the Eastern region of Kenya prior to introduction and implementation of rotavirus vaccination into the national immunization programmes.
Study design: During the period September 2009 through August 2011, 500 stool samples were collected from children < 5 years of age admitted for acute diarrhoea in hospitals in the Eastern region of Kenya, and analysed for the presence of group A rotavirus using an enzyme immunoassay. G and P genotypes of these rotaviruses were determined using nested reverse-transcriptase polymerase chain reaction. **Results**: One hundred and eighty nine (38%) of the samples analysed were positive for rotavirus. The following G types were detected: G9 (64%), G8 (13%), G12 (8%), G1 (4%), and G2 (1%). Mixed G types were also detected in 2 samples (2%), and 9 samples (8%) were non-typeable. The following P types were detected: P[8] (47%), P[4] (17%), and P[6] (7%). A mixed P type was found in 11% of samples, and 19% were non-typeable. The most dominant strain was G9P[8] (40%), followed by G8P[4] (10%) and G12P[6] (5%). Other G and P type combinations detected were G1P[8] (4%), G12P[4] (4%), G9P[6] (3%), G9P[4] (2%), G8P[8] (2%), and G2P[4] (1%). **Conclusions**: The present study divulges the recurring changing genotypes of rotavirus circulating in Kenya with genotypes G9 and G8 being the dominant rotavirus strains circulating in the Eastern region of Kenya between 2009 and 2011. Additionally, the emerging, uncommon G12 genotype was detected for the first time in Kenya.

Poster 41:

DIAGNOSTICS AT POINT OF CARE IN ETHIOPIA

OLI ABATE

Hawassa University, Ethiopia

My medical practice involves evaluating, diagnosing and caring for patients of varying medical illness coming from the southern part of the country. Due to lack of standard laboratory and other diagnosing investigations, most of my patients get subjected to empiric therapy which involves administration of multiple drugs with considerable side effects and interaction. An opportunity to upscale my knowledge and skill regarding point of care diagnostic investigations will go a long way in improving the quality of possible care I can deliver to my patients. In addition, my work as a lecturer entails providing clinical training to health officer students who, after graduation, will practice medicine in the most resource constrained parts of the country. The skill from the workshop will also improve the standard of the education I will provide to my students.

Poster 42:

DIAGNOSTICS FOR CEREBRAL AND PLACENTAL MALARIA

Onkoba Nyamongo

INSTITUTE OF PRIMATE RESEARCH, Kenya

I am currently involved in malaria research in respect to developing the non human primate as human model for malaria in pregnancy and cerebral involvement. Determining host parasite interactions, parasite biology, malaria and helminthes coinfection in respect to parasite load and immunological response interactions and preclinical evaluation of blood stage candidate vaccines. Also testing the anti-plasmodial and therapeutic activity of selected medicinal herbs and old drugs as a cheap remedy to malaria. In malaria in pregnancy and cerebral involvement we have a keen interest in identifying molecules that can be used as prototype diagnostic target which are non invasive in human diseases.

Poster 43:

ANALYSIS OF *SCHISTOSOMA MANSONI* CANDIDATE ANTIGENS AS DIAGNOSTIC TARGETS FOR SCHISTOSOMIASIS

Ogongo P[1] , Wilson RA[2] and Kariuki TM[1]

[1]Institute of Primate Research, P.O Box 24481 00502, Karen, Nairobi, Kenya;
[2]Department of Biology, University of York, Heslington, York, United Kingdom.

Demonstration of schistosome eggs in stool/urine is the gold standard for clinical examination of schistosomiasis. These tests have low sensitivity making infections undetectable. Antibody based assays cannot distinguish between past and active infections thus unsuitable for follow up after drug administration. Molecular techniques have high specificity and sensitivity but are expensive and unavailable at the point of care centers. Bioinformatics and Proteomics approaches can be used to identify and characterize schistosome proteins. These proteins originate from parasite life cycle stages and released into the bloodstream and/or urine, derived from worm gut, worm tegument, egg secretions or released products of dead eggs thus good diagnostic targets. Three schistosome proteins were identified, Cathepsin B, Asaparaginyl endopeptidase) and Sm200. Using these approaches, peptide sequences from these proteins synthesized as multiple antigenic peptides were used to immunize rats. Serum from immunized rats were used to test the suitability of these targets using Enzyme Assays, Blot Assays and Immunocytochemistry with worm sections. Results show that multiple antigenic peptides raised antibodies in rats making these proteins possible diagnostic targets that can be developed further to assess their sensitivity aiming at coming up with an assay capable of detecting the lowest number of worms in the host.

Poster 44:

WESTERN PROVINCE (KENYA) EXTERNAL QUALITY ASSESSMENT SCHEME

Paul Rajula[1], Francis Wesiela[2], Lucy Mwikali[2], Ben Weimi[3]

[1] Ministry of Medical Services
[2] Medical Services – GOK
[3] EGPAF-Kisumu

Western Province External Quality Assessment Scheme (WEPEQAS) is performed in the Western Province of Kenya. The scheme is a project that is run by government of Kenya medical laboratory staff supported by APHIA-Plus. The scheme involves results comparison of CD4 and hemoglobin (HB) across machines in the province. The objective of the scheme is to ensure there are accurate and reliable results produced by the CD4 and HB machines in the province. Proficiency test panels are prepared in the provincial headquarters and sent to 17 laboratories in the province. The results are then sent back and analysis is done. The comparison and scoring are done by the use of Mean; Standard deviations and coefficient of variations. There is relatively little variation in regard to different machines platforms in performance. Since 2009, the 17 CD4 and HB machines assessed proved that. However a 5 percent variation should be allowed. Use of point-of-care machines serves the same results as high output and big on-the-bench machines. The former however stands better chance for patient access to services.

Poster 45:

MANUALLY ACTUATED MICROFLUIDIC DEVICE FOR BLOOD PLASMA SEPARATION

Paul Scanlan, Farid Amalou and Wenmiao Shu*

School of Engineering and Physical Sciences, Heriot-Watt University, Edinburgh, United Kingdom

We have developed a simple and low-cost microfluidic device for blood plasma separation that can be operated simply by using the finger. In order to detect disease biomarkers from a whole blood sample, high-efficiency blood plasma separation is required.

Conventional methods such as centrifugal and filtration can be expensive and time consuming, which has limited its usages in the field, at home or for resource limited applications. This has led to the development of microscale separation techniques [1], some based on the afore-mentioned principles [2-4] and others in microfluidics. Although several types of microfluidic device have been demonstrated, most approaches rely on high precision microchannels the addition of chemicals or a driving force from an external syringe or vacuum retreatment [5-9]. We developed a novel device that is able to efficiently separate plasma from blood using a simple, one-step, manual process, incorporated into a single chip. The device is low-cost and desirable for low-resource, point-of-care diagnostics.

The chip works using the fact that red blood cells carry a greater density than that of white blood cells and the plasma which make up a whole blood sample [10]. As blood flows through the chip, the effects of gravity draw red blood cells to the bottom of a series of trenches, separating them from the rest of the sample. In order for this to work effectively, it is vital to regulate the rate of flow of blood through the chip [11] and our design uses an applied external pressure over an elastic membrane to create the driving pressure.

In our practical demonstration we will show how the 3-dimensional chip is fabricated and assembled from plastic sheets and adhesive transfer tape. The design of each individual layer can be created using a low-cost CO2 high power laser. The simple fabrication process allows the creation of complex 3-D multi-layer structures where each layer defines the inlet/outlet chambers, micro-channels and separation trenches.

Following assembly of the chip, we will go on to demonstrate it in operation by using it to separate a whole sample of animal blood. Similar tests have already been carried out to determine the efficiency of the device, the results of which can be seen in the diagrams in Figure 1. To the visible eye, there is a clear separation between the red blood cells (from sheep) trapped in the separation trench and the clear plasma found in the outlet chambers. Under microscope it is possible to see almost no residual cellular traces found in the extracted plasma, indicating a blood separation efficiency close to 100%.

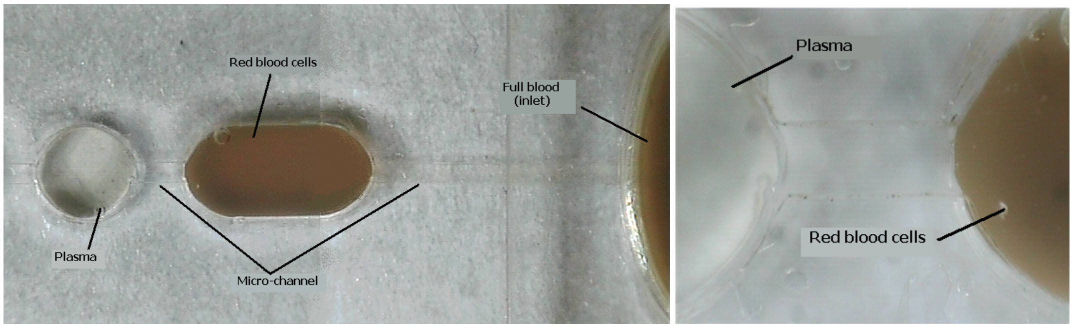

Figure 1 – Blood Plasma Separation Test

1. ***Kersaudy-Kerhoas M, Dhariwal RS, Desmulliez MPY (2008)*** *Recent advances in microparticle continuous separation. IET Nanotechnobiol. 1:1–13*
2. ***Brenner T, Haeberle S, Zengerle R, Ducree J*** *(2004) Continuous centrifugal separation of whole blood on a disk. In: Proceedings of eighth international conference on miniaturized systems for chemistry and life sciences, pp 566–568*
3. ***Thorslund S, Klett O, Nikolajeff F, Markides K, Berquist J*** *(2006) A hybrid poly(dimethylsiloxane) microsystem for on-chip whole blood filtration optimized for steroid screening. Biomedical Microdevices Vol. 8: 73-79*
4. ***Crowley TA, Pizziconiin V*** *(2005) Isolation of plasma from whole blood using planar microfilters for lab-on-a-chip applications. Lab on a Chip, 5(9):922-9*
5. ***Faivre M, Abkarian M, Bickraj K, Howard S*** *(2006) Geometrical focusing of cells in a microfluidic device: an approach to separate blood plasma. Biorheology 43:147–159*
6. ***Yang X, Forouzan O, Brown TP, Shevkoplyas SS****, (2012) Integrated separation of blood plasma from whole blood for microfluidic paper-based analytical devices, Lab Chip, 12, 274-280*
7. ***Kersaudy-Kerhoas M, Kavanagh DM, Dhariwal RS, Campbell CJ, Desmulliez MPY****, (2010) Validation of a blood plasma separation system by biomarker detection, Lab Chip, 10, 1587-1595*
8. ***Jaggi RD, Sandoz R, Effenhauser CS (2007)*** *Microfluidic depletion of red blood cells from whole blood in high-aspect-ratio microchannels. Micro NanoFluid 3:47–53*
9. ***Dimov IK, Basabe-Desmonts L, Garcia-Cordero JL, Ross BM, Ricco AJ and Lee LP*** *(2011), Stand-alone self-powered integrated microfluidic blood analysis system (SIMBAS). Lab Chip, 11, 845-850*
10. ***Fung YC (2004)*** *Biomechanics, 2nd edn. Springer, New York*
11. ***Abkarian M, Viallat A (2008)*** *Vesicles and red blood cells in shear flow. Soft Matter 4:653–657*

Poster 46:

DIAGNOSIS OF CYCLOSPORIASIS IN NON-HUMAN PRIMATES

Purity Nguhiu

omo Kenyatta University of Agriculture and Technology/ Ministry of Livestock Development, Kenya

I am a PhD student attached at the Institute of Primate Research and working on Clinical Epidemiology of Cyclospora in non-human primates in view a laboratory model for Cyclosporiasis in humans. In view of this, I hope to gain practical knowledge on point of care diagnostics and devices that may be developed /fabricated for rapid diagnosis of Cyclosporiasis.

Poster 47:

CAPILLARY PLATFORM FOR MALARIA DETECTION FROM WHOLE BLOOD BY HYDROGEL PCR

Roshini Samuel[1], Kimberly Martin[2], Brian Taylor[2], Alex Stickel[2], Dammika Manage[2], Rochelle Cruz[1], Stephanie Yanow[1,3]

[1] School of Public Health, University of Alberta, Edmonton, Canada
[2] Department of Oncology, University of Alberta, Edmonton, Canada
[3] Provincial Laboratory for Public Health, University of Alberta, Edmonton, Canada

Background: Malaria kills close to 1 million people annually, mostly young children. Sensitive, affordable, and rapid diagnosis is critical for patient care and to support eradication strategies. Our objective is to modify a standard haematocrit capillary to enable direct testing for malaria by qPCR and melt curve analysis.

Methods: Reagents for malaria real-time PCR are combined with an acrylamide solution within a capillary to form a 'hydrogel'. The hydrogel is desiccated for long-term storage and to enable sample delivery by capillary action. The gel is rehydrated by dipping the capillary in a 1:5 dilution of a clinical sample. Capillaries are run on a real-time PCR instrument that was developed in our lab.

Results: The capillary-based assay detects the four major species of *Plasmodium* directly from unprocessed clinical samples. The limit of detection is 10 parasites/μL of blood. Positive and negative controls were designed to provide quality control for molecular testing in capillaries. We have also shown that hydrogel capillaries can be stored at room temperature for at least 4 weeks with no loss in performance.

Conclusions: Our innovative platform combines sample collection and testing within a single, low-cost, disposable capillary for molecular diagnostics at the point-of-care. Blood can be added to capillaries directly, without requiring sample processing. Capillaries are also adapted for long-term storage in the field obviating the need for a cold chain to preserve molecular reagents. The sensitivity, affordability and user-friendliness of this technology make it an excellent platform for a variety of clinical applications within resource-limited settings.

Poster 48:

USE OF CIRCULATING ANODIC ANTIGEN (CAA) DETECTION AS A SURROGATE MARKER FOR DETERMINING WORM AND EGG BURDEN IN SCHISTOSOMIASIS INFECTIONS

G. J. van dam[1], Ruth Nyakundi[2], Thomas Kariuki[2], P.L.A.M. Corstjens[1]

[1]*Leiden University Medical Center, Netherlands*
[2]*Institute of Primate Research, Nairobi, Kenya*

In human schistosomiasis infections, it is impossible to directly determine worm burden and hence surrogates must be used. In this study we looked at the relationship between worm burden and circulating anodic antigen (CAA) concentrations in serum of baboons infected with *S. mansoni* . Four groups of baboons (n=2) were infected with 5, 25, 100 and 500 *S. mansoni* cercariae. 10 weeks post infection, the baboons were perfused and worm burden quantified. Faecal egg output was determined by the Kato Katz technique and microscopy and serum was collected for antibody and CAA determination. CAA levels were measured with the UCP-CAA LF assay that uses the principle of lateral flow through a nitrocellulose strip on which the presence of CAA in a sample is shown by the capture with anti-CAA monoclonal antibodies and visualization with anti-CAA Ab-UCP conjugates. The immobilized UCP is shown by scanning the the strips with a portable lightweight UCP reader. The lower level of detection using 20 µl serum was 30 pg CAA/ml, and 1 pg/ml with 500 µl serum The latter approach involved concentration of the TCA-treated sample and allowed demonstration of an infection with 1 worm per baboon. Also a clear correlation of CAA levels with worm numbers was shown. In addition, using the same techniques, detection of CAA in urine from humans was shown to be highly sensitive and specific, both for *S. haematobium* and *S. mansoni* infections. With concentration of 4 ml of TCA-treated urine, even 100% sensitivity was shown in samples with very low infection levels as measured by multilple egg counts and PCR. Also the CAA levels in urine correlated significantly with *S.h.* and *S.m.* egg loads.

Poster 49:

DEVELOPMENT OF MONOCLONAL ANTIBODIES FOR USE IN DIAGNOSIS OF RIFT VALLEY FEVER VIRUS

Ashur Salame

JKUAT-INTROMID

My studies involve the development of monoclonal antibodies for use in diagnosis of Rift Valley Fever Virus, and my future plan is to avail this diagnosis assay at a point of care. Attending the workshop will grant me an opportunity to interact with scientists who are experienced in this field and I will gained techniques that will be applicable and of great importance to my study.

Poster 50:

MOLECULAR CHARACTERIZATION OF ANTIMICROBIAL RESISTANCE ON ENTEROBACTERIACEAE FROM SEVERELY MALNOURISHED CHILDREN ATTENDING MBAGATHI DISTRICT HOSPITAL

Samuel Mwangi Njoroge

Jomo Kenyatta University of Agriculture and Technology/ KEMRI

Diarrhoea in children under five years, continue to be a major public concern in developing countries like Kenya. Malnutrition can predispose a child to diarrhoea or be a result of persistent diarrhoea. The study design will be cross-sectional in nature and will be nested in a bigger cotrimoxazole prophylaxis clinical trial. The study site is Mbagathi District Hospital and will target the urban-poor population from Kibera. Study sample size will be 217 rectal swabs/ stool samples from severely malnourished children with < -3.0 standard deviation from mid upper arm circumference. The study seeks to identify etiologic agents of diarrhoea and equally important, their drug resistance patterns towards amino-penicillins cephalosporins and sulfonamide. Rectal swabs/Stool will be cultured on MacConkey and Salmonella Shigella Agar. Lactose fermenters colonies will also be screened for extended beta-lactamases, sulfomnamide, and fluroquinolone resistance using a 16 drug panel. Resistant isolates will be screened for extended beta-lactamase genes, dehydrofolate reductase genes or fluoroqinolone resistance genes using qualitative multiplexPCR. Confirmatory test will be done by nucleotide sequencing and comparison made with strains on GeneBank. The project is estimated to cost 1062850 Kenyan shillings with a nine month time-frame. Supervisors will be involved in the management of resources. Findings will be published.

Supervisors are Dr. John Kiiru and Dr. Gideon Kikuvi all who have published some work on antimicrobial drug resistance. Dr John is a scientist at KEMRI-center for microbiology research while Dr Gideon is a lecturer/ Deputy Director JKUAT/ITROMID.

Poster 51:

MOLECULAR CHARACTERIZATION OF THE RANTES GENE POLYMORPHISMS IN NAIROBI PROVINCE, KENYA; POTENTIAL FOR PERSONALIZED MEDICINE

Shem Mutuiri[1], Samoel A. Khamadi[2], Helen L. Kutima[4] , Lamech M. Mwapagha[4], James K. Munyao[4], Irene Wanjiru[5]

[1]*Institute of Primate Research (IPR), Jomo Kenyatta University of Agriculture and Technology, Kenya*
[2]*Center for Virus Research, Kenya Medical Research Institute, Kenya*
[3]*Department of Zoology, Jomo Kenyatta University of Agriculture and Technology*
[4]*Institute of Tropical & Infectious Diseases, Jomo Kenyatta University of Agriculture and Technology*
[5]*Kenyatta University*

The objective of the study was to determine the existence and distribution of polymorphism in RANTES gene alleles in HIV positive and HIV negative controls (blood donors) in Nairobi Province, Kenya. METHODS: We determined the existence and distribution of RANTES gene Single Nucleotide Polymorphisms (SNPs) using Polymerase Chain Reaction (PCR) and Restriction Fragment Length Polymorphism (RFLP) in HIV positive persons (n=100) and HIV negative controls (n=100). RESULTS: We found that the RANTES gene polymorphisms were existent in the Nairobi population. We found that the two most common RANTES promoter compound genotypes, G1 (403G/G, 28C/G) found in 19% of HIV positive persons and 13% of HIV negative blood donors and G4 (403G/A, 28C/C) found in 50% of HIV positive persons and 54% of HIV negative blood donors, were associated with altered risk of HIV transmission and progression. There was no statistical difference in the distribution of RANTES gene polymorphisms (P=0.092). CONCLUSIONS: These results indicate that the RANTES gene polymorphisms that are a risk factor for HIV transmission and as a protective factor for HIV progression are existent in the Nairobi population in Kenya. It calls for further associative studies that can result in the incorporation of RANTES analogues or antagonists in HIV/AIDS therapy in Kenya.

Poster 52:

NOSTIC TOOL FOR DETECTION OF ENTEROPATHOGENIC BACTERIA AND PROTOZOA IN WATER

<u>Kiruki S.</u>

Egerton University , Njoro

Waterborne diarrhoeal diseases are responsible for a huge proportion of morbidity and mortality in developing countries. Most common pathogens include E. coli, Hafnia alvei (eae), V. cholerae & Aeromonas spp., Salmonella spp., G. lamblia and E. histolytica. The high prevalence of undiagnosed diarrhoea at Njoro Health Centre and Nakuru municipality is linked to polluted waters and the culprits pathogens are E. histolytica (10%), G. lamblia (2%), S. typhi (27%) and other parasites (12%). The aim of this study bid to develop a diagnostic tool that can be used routinely to detect the presence of enteropathogenic bacteria and protozoa in water.

Poster 53:

A SIMPLE, CHEAP AND PORTABLE DEVICE FOR POINT-OF-CARE DIAGNOSIS OF SLEEPING-SICKNESS

Stefan H. Holm, Jason P. Beech, and Jonas O. Tegenfeldt

Lund University, Solid State Physics
Box 118, SE-221 00 LUND, Sweden

Sleeping sickness (SS) is a fatal disease caused by a protozoa of the Trypanosoma brucei subgroup.1 The tsetse fly vector injects the parasite while feeding on blood. Initially the parasites proliferate in the hemolymphatic system of infected patients and then spread to the central nervous system (CNS) where they cause increasing neurological dysfunction. Treatment of SS includes a multitude of side effects. In addition, if the parasite is given enough time to invade the CNS, treatment relies to a great extent on Melarsoprol, a highly toxic arsenic derivate with a mortality rate of 5%. This emphasizes the need for a method of early detection with a minimum level of false positives.
Accurate detection requires finding the actual parasite, most often done by simple microscopic examination of blood smears, a very time-consuming method with a low detection limit. Here, a key bottleneck is the low concentration of the parasite. Current alternative methods are expensive and inadequate for use in the field.2 Our method provides a dramatically simpler and cheaper alternative with the potential of enabling a fast and cheap point-of-care device with significant impact on the diagnosis of sleeping sickness.
Our microfluidic devices rely on Deterministic Lateral Displacement (DLD)3 which our group has shown can be employed to separate particles based on shape4 and deformability5. In the present work we report a simple DLD device, driven simply by hand with a syringe, with which we can analyze several microlitres per minute, resulting in a time per test of ~10 min.

1. M. P. Barrett, R. J. S. Burchmore, A. Stich, J. O. Lazzari, A. C. Frasch, J. J. Cazzulo and S. Krishna, The Lancet, 2003, 362, 1469-1480.
2. F. Chappuis, L. Loutan, P. Simarro, V. Lejon and P. Buscher, Clin. Microbiol. Rev., 2005, 18, 133-146.
3. L. R. Huang, E. C. Cox, R. H. Austin and J. C. Sturm, Science, 2004, 304, 987- 990.
4. S. H. Holm, J. P. Beech, M. P. Barrett and J. O. Tegenfeldt, Lab on a Chip, 2011, 11, 1326-1332.
5. J. P. Beech, S. H. Holm, K. Adolfsson and J. O. Tegenfeldt, Lab on a Chip, 2012, 12.

Poster 54:

ANALYTICAL CHEMISTRY FOR DIAGNOSIS AND CHEMOTHERAPY

Swaleh Sauda

Kenyatta University

I undertake research in natural product chemistry as well as applied analytical chemistry.

- Isolation, biological investigation and structural elucidation of the secondary metabolites from Kenyan medicinal plants that exhibit antimicrobial and antifungal activities, cytotoxicity effects and wound healing properties.
- Investigation of Natural products with antimalarial, trypanocidal and leishmanicidal activities and efficacy of medicinal plants
- Chemical profile analysis of Essential oils and determination of their antimicrobial activities.
- Bioavalaibility of Vitamin A, E and C in people living with AIDS ad effects of indigenous foods in the management of HIV and AIDS.
- Quantification of phytochemicals with antioxidant properties from variety of fruits found in selected areas of Kenya.
- Cyanogenic potential levels of cassava cultivars from different Ago-ecological zones in Kenya.

Poster 55:

TB DIAGNOSTICS AT POINT OF CARE IN ZIMBABWE

Tendai Gadzikwa

University of Zimbabwe, Zimbabwe

I have just recently joined the Chemistry Department at the University, and I am in the process of developing a research program that is feasible with our limited resources. As I am new to the area of diagnostics, I believe that the Point-of-Care Diagnostic Workshop will be an invaluable opportunity for me to learn from those with some experience in the field already. I also hope to develop relationships with other African scientists, and perhaps initiate regional collaborations with them. I am embarking on a collaboration with the Gibbs-Davis group at the University of Alberta. They have developed a DNA detection method based on DNA amplification, which they intend to apply to the detection of TB. My role in this partnership will be to develop a protocol for this method; one that an be implemented easily in labs and clinics in Africa. Dr. Julianne Gibbs-Davis will be demonstrating the amplification/detection method at the workshop.

Poster 56:

SIMPLE TECHNOLOGIES FOR TESTING FOR FAKE DRUGS

Thomas Musembi

Sidai Africa Ltd

Sidai Africa Ltd is a newly established social enterprise in Kenya that seeks to offer quality veterinary services and products to livestock keepers, especially the small scale and marginalized ones. A major challenge of offering this service is the unavailability of simple penside diagnostic techniques which would go a long way in improving the quality service we desire. I am particularly interested in simple technologies that can be used in testing for fake drugs that are rife in Kenya especially in the veterinary sector which hardly gets any support from regulatory authorities.

Poster 57:

THE ROLE OF REGULATORY T-CELLS IN PATHOGENESIS OF TRYPANOSOMIASIS IN MOUSE MODEL

Victor Alioni[1], Rasheed Khalid[2], Gerald Areba[2]

1. National Livestock Resources Research Institute (NaLIRRI)/ NARO- UGANDA and Department of Biochemistry and Molecular Biology, Egerton University, Njoro Campus- KENYA.
2. Department of Biochemistry and Molecular Biology, Egerton University, Njoro Campus, Nakuru-KENYA

African trypanosomiasis is a fatal infection of man and animals. It is often diagnosed as early and late stages based on clinical presentation. Infection with African trypanosomiasis invokes both innate and adaptive immune responses from the host. Macrophages elicited from trypanosome-infected hosts exhibit increased expression of pro-inflammatory and immune-regulatory molecules with the initial inflammatory response being beneficial to the host at early stages of infection, however, a sustained inflammation can cause pathology. Limitation of aberrant immune responses and maintaining tolerance toward self tissues is required and is the function of Regulatory T cells (Tregs). Although Tregs have been implicated in infectious diseases, particularly in chronic or persistent infections where they may prevent immunopathology, but also compromise pathogen eradication through manipulating antigen presenting dendritic cells (DC), and thus reducing long-lasting interactions between effector T cells and DC, induction and mode of action of the various distinct Treg subsets remain ill defined particularly in Trypanosomiasis. This study hypothesizes that T-regulatory (Tregs) cells do not play a functional role if at all expressed, in the severity and clinical course of rhodesiense type of trypanosomiasis in mice. The study will determine and profile expression of pro-inflammatory and T-regulatory cells in the course of trypanosomiasis and to determine the effect of T-regulatory cell depletion on the pathogenesis of trypanosomiasis in Swiss white mice. A *Trypanosoma brucei rhodesiense* strain from KARI-TRC trypanosome bank will be transfected with a construct containing the gene for green fluorescent protein (GFP) under the control of the metacyclin promoter and the GFP prepared inoculums of 4 x 103 µl-1 be introduced into Swiss white mice. Fluorescent microscopy will be used to identify the parasitemia levels in peripheral blood at intervals of 72 hours four days post infection and in contrast immune cells expressed four days post infection, here-in considered as early stage will be sampled from blood. The late stage will sample from both brain and blood tissues to be analyzed by flow Cytometry and results compared with the control. This study hopes to identify novel biomarkers associated with progression of trypanosomiasis which should bolster existing diagnostic approaches and should enhance accuracy in detection for effective treatment of the disease. Findings from this study should offer opportunities for identification of possible vaccine targets or identify options for immunotherapy.

Poster 58:

VALIDATION OF VARIABLE SURFACE GLYCOPROTEIN (VSG) FOR DIAGNOSIS OF AFRICAN TRYPANOSOMES

Vincent Ouma, Adung`a VA, Masiga DK

Department of Biochemistry and Molecular Biology, Egerton University, P.O. Box 536, Egerton, Kenya, Molecular Biology and Biotechnology Unit, International Centre of Insect Physiology and Ecology (ICIPE), P.O. Box 30100-00100, Nairobi, Kenya.

Trypanosoma brucei is a tsetse transmitted flagellated parasitic protozoon that causes African trypanosomiasis, a disease affecting man and animals. Two subspecies of *T. brucei, T. b. rhodesiense* and *T. b. gambiense* cause acute and chronic forms of Human African trypanosomiasis (HAT) respectively. In mammalian hosts, *T. brucei* evades immune clearance by antigenic variation, the sequential expression of antigenically distinct glycosylphosphatidylinositol (GPI)-anchored variable surface coats called variable surface glycoprotein (VSG). The variable antigen types (VATs) in distinct VSGs are expressed in a reversible and hierarchical fashion, hence are potential candidates for diagnosis. Here, using monkey model, VATs expressed early on infection with T. b. rhodesiense were determined and recombinant proteins expressed and purified using bacterial expression system. Preliminary screening show that a set of these VATs is recognized by infected human serum samples from East African region, and are present in the VSG gene repertoire. Therefore, these VATs are potential candidates for development of a serodiagnostic kit.

Poster 59:

POPULATION GENETICS OF HOST AND PARASITE GENES INVOLVED IN INVASION OF MALARIA

Virginia Gichuru

Strathmore University

My research is on population genetics of host and parasite genes involved in invasion of malaria. Strathmore University, has recently opened a medical clinic and is interested in becoming a lead player in providing adequate and excellent medical care in the region.

Poster 60:

A COMPARATIVE ANALYSIS OF DUAL AND NO-CHOICE REPELLENCY BIOASSAYS OF ESSENTIAL OIL OF *TAGETES MINUTA* AGAINST CLIMBING RESPONSE BEHAVIOR OF *RHIPICEPHALUS APPENDICULATUS*

W. Wanzala1,2j, R.W. Mukabana2,4, W. Takken5, A. Hassanali2,3

[1]*School of Pure and Applied Sciences, Department of Biological Sciences, South Eastern University College (a constituent college of the University of Nairobi), P.O. Box 170-90200, Kitui, Kenya.,*
[2]*Behavioural and Chemical Ecology Department (BCED), International Centre of Insect Physiology and Ecology (ICIPE), P.O. Box, 30772-00100, Nairobi, Kenya.,*
[3]*School of Pure and Applied Sciences, Kenyatta University, P.O. Box 43844-00100 GPO, Nairobi, Kenya.,*
[4]*School of Biological Sciences, University of Nairobi, P.O. Box 30197-00100, Nairobi, Kenya.,*
[5]*Wageningen University and Research Centre, Laboratory of Entomology, P.O. Box 8031, 6700 EH Wageningen, the Netherlands.*

The study compared two sets of bioassays designed to evaluate repellency of essential oil of *Tagetes minuta* against climbing response behaviour of adult, *Rhipicephalus appendiculatus*, the vector of deadly livestock disease, East Coast fever. The study aimed at evaluating the appropriate bioassay set up suitable for screening repellent essential oils that may become applicable in preventive measures for managing arthropod vectors and vector-borne diseases. All bioassays were conducted under the same laboratory conditions and time. In both bioassays, repellency was dose-dependent and significance differences between doses remained the same at $P<0.0001$, ($n = 5$). However, for the same doses, mean percent repellence was lower in the no-choice bioassay (ranging from 17.88±2.39% to 96.5±1.88%) than in the dual-choice bioassay (ranging from 57.92±7.11% to 100.00%). This difference was significant (t (0.05) = 3.256; $P = 0.047$) but its underlying mechanism however, remained unknown. Probit analysis showed that to achieve the same repellent effect, a higher repellent dose is required in no-choice bioassay than in a dual-choice bioassay, hence the former proving unsuitable for screening purposes. These choice bioassays provide baseline data against which novel tick repellents/attractants may be evaluated for development into agents suitable for providing prophylactic measures in integrated pest management.

Summary of Participants

country	location	participants
Kenya	Aga Khan University Hospital	1
USA	Boston University	2
Kenya	Bungoma District Hospital	1
USA	California Polytech State University	1
South Africa	Council for Scientific & Industrial Research	1
USA	Diagnostics For All	1
Kenya	Egerton University	9
Switzerland	Foundation for Innovative New Diagnostics	1
Switzerland	Hackteria	1
Ethiopia	Haramaya University	1
USA	Harvard University	2
UK	Heriot Watt, Edinburgh	2
Kenya	Institute of Primate Research	22
Kenya	International center for insect physiology	2
Kenya	International Livestock Research Institute	3
Rwanda	ISAE (Institut Supérieur d'Agriculture et d'Elevage)	1
Uganda	Joint Clinical Research Center	1
Kenya	Jomo Kenyatta University of Agriculture and Technology	4
Kenya	KEMRI (Kenya Medical Research Institute)	5
Kenya	Kenya Polytechnic	3
Kenya	Kenyatta University	5
Canada	King's University College	1
USA	Lawrence Livermore National Laboratory	3
Sweden	Lund University	3
Uganda	Makerere University	6
Canada	McMaster University	2
Kenya	Moi University	1
Kenya	MoLD (Ministry of Livestock development)	1
Kenya	MoMS (Ministry of Medical Services)	1
Kenya	Mt Kenya University	1
Kenya	Neema Healthcare Services	1
Kenya	National Quality Control Laboratory	1
Kenya	National Museums Kenya	1
USA	Purdue University	1
Canada	SENTINEL	1
Kenya	SEUCO (South Eastern University College)	1
Kenya	Sidai Africa (NGO)	1
Kenya	Strathmore University	1
Tanzania	Tsetse & Trypanosomiasis Research Institute (TTRI / EGFAR)	3
Canada	University of Alberta	7
USA	University of California, Irvine	1
UK	University of Glasgow	2
Kenya	University of Nairobi	7
USA	University of Notre Dame	2
UK	University of Oxford	1
Brazil	University of Sao Paulo	1
UK	University of Southampton, UK	2
USA	University of Washington	1
USA	University of Wisconsin-Madison	1
Zimbabwe	University of Zimbabwe	1
Kenya	Vetlabs Nairobi	1

List of Participants and Presenters

Maina, Daniel	Aga Khan University Hospital
Klapperich, Catherine	Boston University
Linnes, Jacqueline	Boston University
Atema, Erick	Bungoma District Hospital
Martinez, Andres	California Polytech State University
Govindasamy , Klariska	Council for Scientific & Industrial Research
Mace, Charles	Diagnostics For All
Ombira, Jacktone	Egerton
Adunga, Vincent	Egerton University
Alioni, Victor	Egerton University
Gerald, Areba	Egerton University
Kamanyi, Kevin	Egerton University
Kiruki, Silas	Egerton University
Mavura, Hawa	Egerton University
Obonyo, Meshack	Egerton University
Othira, Jacktone	Egerton University
Owino, Vincent	Egerton University
Ndung'u, Joseph	Foundation for Innovative New Diagnostics
Dusseiller, Marc	Hackteria
Bayleyegn, Gizachew	Haramaya University
Kumar, AJ	Harvard University
Thuo, Martin	Harvard University/Kenyatta University
Scanlan, Paul	Heriot Watt, Edinburgh
Shu, Will	Heriot Watt, Edinburgh
Ichagichu, Maina	Institute of Primate Research
Jeneby, Maamum	Institute of Primate Research
Kagira, John	Institute of Primate Research
Kariuki, Tom	Institute of Primate Research
Kinyanjui, Chris	Institute of Primate Research
Kiulia, Nicolas	Institute of Primate Research
Kunyera, Robert	Institute of Primate Research
Maloba, Fredrick	Institute of Primate Research
Mareri, Peter	Institute of Primate Research
Mbaruk, Suleman	Institute of Primate Research
Mutuiri, Shem	Institute of Primate Research
Ndung'u, Michael	Institute of Primate Research
Ngotho, Maina	Institute of Primate Research
Nyachieo, Atunga	Institute of Primate Research
Nyakundi, Ruth	Institute of Primate Research
Nyamongo, Onkoba	Institute of Primate Research
Ochola, Lucy	Institute of Primate Research
Ogongo, Paul	Institute of Primate Research
Onditi, Faith	Institute of Primate Research
Ozwara, Hastings	Institute of Primate Research
Taracha, Evans	Institute of Primate Research
Waititu, Ken	Institute of Primate Research
Fischer, Anne	International center for insect physiology
Fevre, Eric	International Livestock Research Institute
Jores, Joerg	International Livestock Research Institute

Ngatiri, Nimmo	International Livestock Research Institute
Bigirimana, Vincent de Paul	ISAE, Rwanda
Kasambula, Lordwin	Joint Clinical Research Center
Karanja, Simon	Jomo Kenyatta University of Agriculture and Technology
Maina, Naomi	Jomo Kenyatta University of Agriculture and Technology
Njoroge, Samuel Mwangi	Jomo Kenyatta University of Agriculture and Technology
Muthinja, Julianne	Jomo Kenyatta University of Agriculture and Technology /ICIPE
Ochola, Elizabeth	KEMRI-KSM
Ashur, Salame	KEMRI-Nairobi
Ole Kwallah, Allan	KEMRI-Nairobi
Waitumbi, John	KEMRI-Walter Reed
Aluoch, Austin	Kenya Polytechnic
Henry, Kenji	Kenya Polytechnic
Ndunda, Theresia	Kenya Polytechnic
Kipchumba, Kogo Kenneth	Kenyatta University
Kiprono, Towett Gideon	Kenyatta University
Maranga, Dawn	Kenyatta University
Sauda, Swaleh	Kenyatta University
Swaleh, Sauda	Kenyatta University
Vanderveen, Jesse	King's University College
Beer, Reg	Lawrence Livermore National Laboratory
Farquar, George	Lawrence Livermore National Laboratory
Vitalis, Beth	Lawrence Livermore National Laboratory
Beech, Jason	Lund University
Holm, Stefan	Lund University
Tegenfeldt, Jonas	Lund/Gothenburg University
Bakuneeta, Chris	Makerere University
Lonzy, Ojok	Makerere University
Matovu, Erick	Makerere University
Mugasa, Claire	Makerere University
Nalunkuma Kazibwe, Anne	Makerere University
Okuni, Julius	Makerere University
Khan, Madiha	McMaster University
Sicard, Clememce	McMaster University
Patel, Kirtika	Moi University
Bor, Mark	Ministry of Public Health and Sanitation
Kiai, Mwangi	MoLD
Rajula, Paula	MoMS
Gitaka, Jesse	Mt Kenya University
Mbithi, Richard	Neema Healthcare Services
Githui, Kem	NMK (National Museums of Kenya)
Maina, Mercy	Purdue University
Rosenberg, George	SENTINEL Bioactive Paper Network
Wanzala, Wycliffe	SEUCO-UON
Musembi, Thomas	Sidai Africa (NGO)
Gichuru, Virginia	Strathmore University
Byamungu, Mechtilda	Tsetse & Trypanosomiasis Research Institute
Lyaruu, Eugene	Tsetse & Trypanosomiasis Research Institute
Malele, Imna	Tsetse & Trypanosomiasis Research Institute
Boadu, Nana Yaa	University of Alberta
Derda, Ratmir	University of Alberta
Gibbs-Davis, Juli	University of Alberta

McKinley, Karen	University of Alberta
Samuel, Roshini	University of Alberta
Serpe, Michael	University of Alberta
Tjhung, Katrina	University of Alberta
Khine, Michelle	University of California, Irvine
Cooper, Jonathan	University of Glasgow
Kremer, Clemens	University of Glasgow
Kimeli, Peter	University of Nairobi
Nasimolo, Johnson	University of Nairobi
Nguta, J.M.	University of Nairobi
Oduma, Jemimah	University of Nairobi
Omondi, Dennis	University of Nairobi
Omondi, Gelliann	University of Nairobi
Wasonga, Caroline	University of Nairobi/KEMRI
Lieberman, Marya	University of Notre Dame
Weaver, Abigail	University of Notre Dame
Morgan, Ruth	University of Oxford
Carrilho, Emanuel	University of Sao Paulo
Morgan, Hywel	University of Southampton, UK
Spencer, Daniel	University of Southampton, UK
Yager, Paul	University of Washington
Weibel, Doug	University of Wisconsin-Madison
Gadzikwa, Tendai	University of Zimbabwe
Hezekiah Chepkwony	National Quality Control Laboratory
Nguhiu, Purity	Vetlabs Nairobi
Maina, Daniel	Aga Khan University Hospital
